# Global trends in research on aging associated with periodontitis from 2002 to 2023: a bibliometric analysis

**DOI:** 10.3389/fendo.2024.1374027

**Published:** 2024-05-10

**Authors:** Xiaomeng Liu, Hongjiao Li

**Affiliations:** Department of Stomatology, Xinhua Hospital, Shanghai Jiaotong University School of Medicine, Shanghai, China

**Keywords:** periodontitis, bibliometric analysis, data visualization, inflammation, aging

## Abstract

**Background:**

Aging has been implicated in many chronic inflammatory diseases, including periodontitis. Periodontitis is an inflammatory disease caused by long-term irritation of the periodontal tissues by the plaque biofilm on the surface of the teeth. However, only a few bibliometric analyses have systematically studied this field to date. This work sought to visualize research hot spots and trends in aging associated with periodontitis from 2002 to 2023 through bibliometric approaches.

**Methods:**

Graphpad prism v8.0.2 was used to analyse and plot annual papers, national publication trends and national publication heat maps. In addition, CtieSpace (6.1.6R (64-bit) Advanced Edition) and VOSviewer (version 1.6.18) were used to analyse these data and visualize the scientific knowledge graph.

**Results:**

The number of documents related to aging associated with periodontitis has steadily increased over 21 years. With six of the top ten institutions in terms of publications coming from the US, the US is a major driver of research in this area. journal of periodontology is the most published journal in the field. Tonetti MS is the most prolific authors and co-cited authors in the field. Journal of Periodontology and Journal of Clinical Periodontology are the most popular journals in the field with the largest literature. Periodontitis, Alzheimer’s disease, and peri-implantitis are current hot topics and trends in the field. Inflammation, biomarkers, oxidative stress cytokines are current research hotspots in this field.

**Conclusion:**

Our research found that global publications regarding research on aging associated with periodontitis increased dramatically and were expected to continue increasing. Inflammation and aging, and the relationship between periodontitis and systemic diseases, are topics worthy of attention.

## 1 Introduction

Periodontitis is an inflammatory disease resulting from infection with periodontal pathogenic microorganisms and dysregulation of the host immune system (1). More than 50% of adults worldwide are affected by different degrees of periodontitis (2, 3). Periodontitis is classified as mild, moderate or severe depending on the degree of inflammation of the disease (4). Severe periodontal tissue damage can lead to aesthetic complications that can be very disturbing to the patient, and new treatment techniques such as laser therapy and digital technology can improve patients’ quality of life (5, 6). Aging underlies the pathogenesis of a range of systemic diseases and has a critical effect on the development of chronic inflammatory diseases (7), such as atherosclerosis and autoimmune (8, 9). Numerous studies have shown a close connection between periodontitis and aging (10–12). During the development of periodontitis, the periodontal tissues are attacked by a large number of free radicals, which intensifies oxidative stress and causes oxidative damage to DNA, consequently accelerating telomere shortening. Shortened telomere length can exert cytotoxic effects, leading not only to disruption of epithelial connective tissue continuity, but also to interference with cell growth and differentiation processes. At the same time, persistent stimulation of bacterial-derived lipopolysaccharide affects cellular senescence of osteoblasts, driving alveolar bone resorption (13, 14). In conclusion, Aging may be one of the key risk factors that promote periodontitis progression.

With time changes as well as functional deterioration, the organism develops a number of chronic and age-related conditions. This process is defined as aging (15). Aging affects normal metabolism as well as promotes the progression of inflammatory responses, disrupting bone remodeling and leading to increased levels of bone resorption (16). In addition, aging impairs the organism’s immune system, mainly through damaging the physiological function of immune cells and weakening the immune effects of biomolecules. This causes a series of processes of immune senescence, immune activation, and inflammatory responses that ultimately result in older adults being more susceptible to autoimmune, and inflammatory diseases. Decreased immune responsiveness with chronic inflammation, which may accelerate the disease process (17–19). Periodontitis is a chronic inflammatory condition related to alterations in the oral microbiota (20). Notably, localized microecological dysregulation in the oral cavity is often caused by a high-inflammatory state in the host (21). Given that aging is related to a low-level “sterile” inflammatory state with no apparent infection, it is suggested that aging may influence the underlying process of periodontitis (22).

Bibliometrics allows for retrospective reviews to discover the relevance of data and make predictions for the future (23). Bibliometrics is a common method of measuring the academic impact of scientific and technical papers, and a means of demonstrating and encouraging emerging scholarship (24). Bibliometrics and visualization analysis can not only effectively integrate information and enhance understanding of research activities, but also analyze research hotspots and future trends in a certain field (25). In recent decades, Bibliometrics has been broadly used in the field of medicine, including obstetrics and gynecology, orthopedics, complementary medicine, and alternative medicine. It has promoted the development of medical research and clinical practice (26). However, the application of bibliometrics in dentistry is still limited at present, and there is a gap in the study of periodontitis related to aging. For this reason, this work intends to systematically sort out the aging research related to periodontitis, summarize the existing research results at home and abroad, assess their academic impact and characteristics, and provide new design ideas for further research.

## 2 Methods and materials

**Figure 1:**
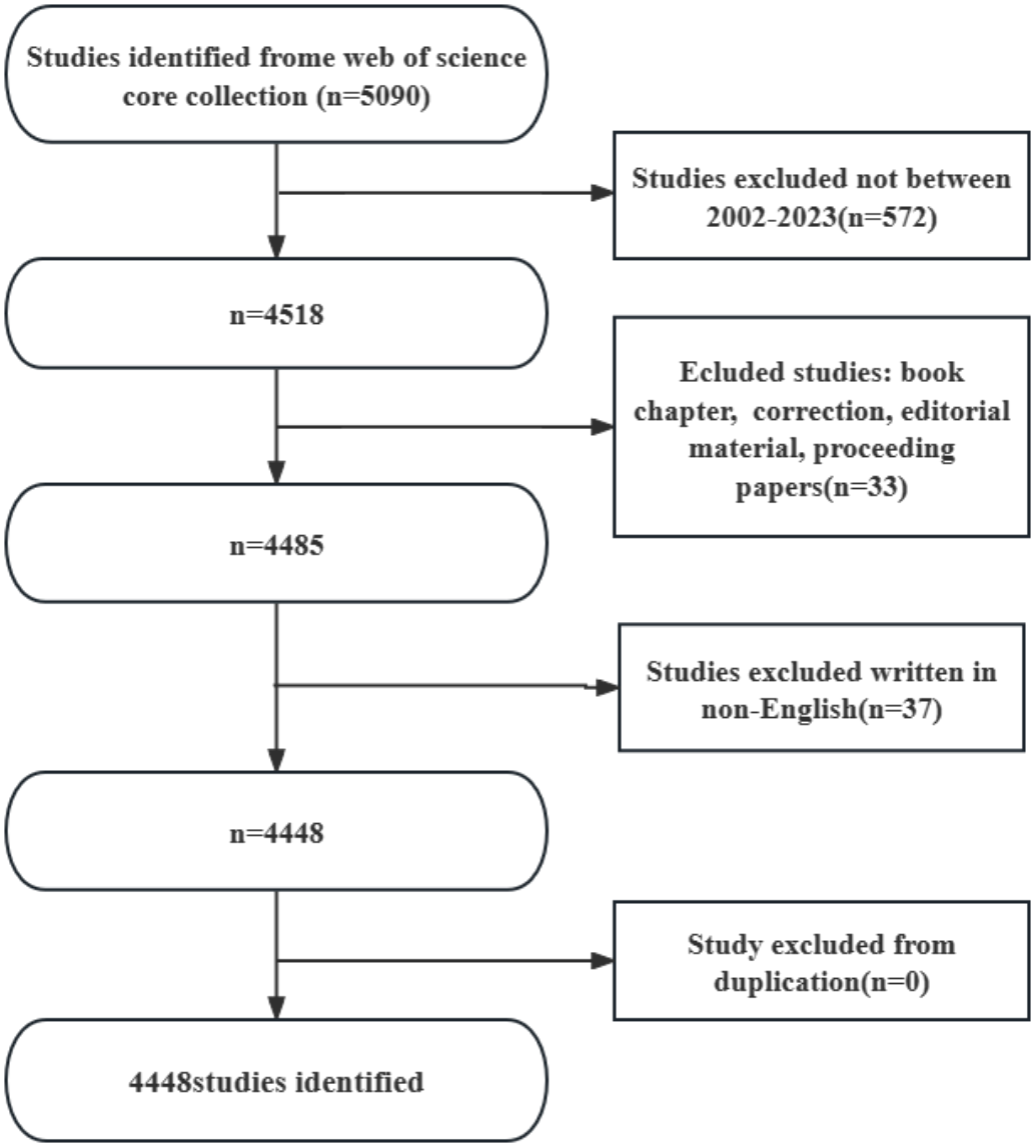
Literature screening flowchart.

Web of Science Core Collection(WoSCC)has better accuracy in labeling literature types than any other database and is considered the best choice for literature analysis, therefore we chose to search in this database. We searched WOS for all articles related to periodontitis and aging from January 1, 2002 to October 25, 2023 with the following search formula (**Figure 1**): (((TS=(aging)) OR TS=(Senescence)) OR TS=(Biological Aging)) OR TS=(Aging, Biological) AND (((TS=(periodontitis)) OR TS=(Periodontitides)) OR TS=(Pericementitis)) OR TS=(Pericementitides) (**Supplementary Table S1**). Literature selection inclusion criteria for this study were as follows: (1) the full text of publications related to periodontitis and aging; (2) the articles and reviews manuscript category were in English; and (3) the article was published between January 1, 2002, and October 25, 2023. The criteria for exclusion were as follows (1) the topic was not related to periodontitis and aging, and (2) the article was a conference abstract, news, or briefing paper. We exported the plain text version of the paper. Graphpad prism v8.0.2 was used to analyze and plot annual papers, national publication trends, and national publication heat maps. In addition, CtieSpace (6.1.6R (64-bit) Advanced Edition) and VOSviewer (version 1.6.18) were used to analyze these data and visualize the scientific knowledge graph. VOSviewer v.1.6.17, created by Waltman et al. in 2009, is a free JAVA-based software for analyzing large amounts of literature data and displaying it in a map format. In order to visualize the results of research in a particular field by mapping the literature co-citation network, Professor Chaomei Chen created the CiteSpace (6.1.6R) software, which envisions the use of an experimental framework for studying new concepts and evaluating existing technologies. This enables users to better understand areas of knowledge, research frontiers and trends, and to anticipate their future research progress.

## 3 Result

**Figure 2:**
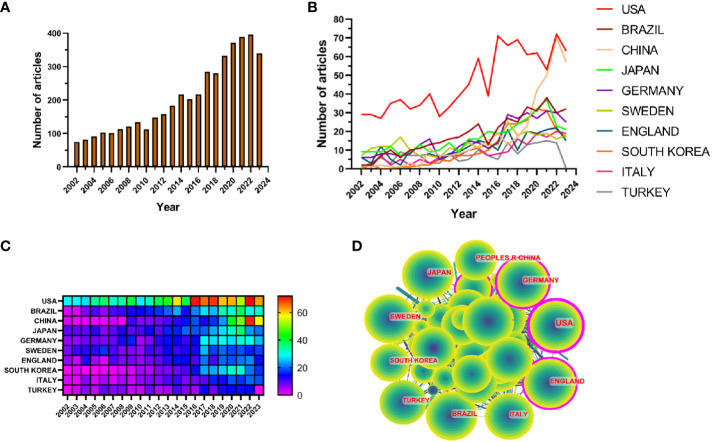
**(A)** Line graph of the volume of communications **(B)** Heat map of national issuances **(C)** Line graph of national issuances **(D)** Map of country cooperation network.

The results showed that from January 1, 2002 to October 25, 2023, the WoSCC database contained a total of 3,240 publications on periodontitis and aging-related literature, including 2,339 (%) articles and 901 reviews (%). The literature covers 120 countries and regions, 3807 institutions and 19263 authors. From 2002 to 2004, the number of articles per year was less than 100, suggesting that the field was not noticed, and after 2005 the number of articles had a yearly increase, which increased rapidly after 2017 and reached the highest value in 2022. It indicates that the correlation between aging and the progression of periodontitis is receiving widespread attention after 2017 (**Figure 2A**).

Research related to periodontitis and aging has been conducted in 120 countries and regions. **Figures 2B, C** show the heat map and line graph of the annual publication volume of the top 10 countries during the past 20 years, and the top 5 countries with the highest publication volume in this field are the United States, Brazil, China, Japan, and Germany. The U.S. accounts for 23.02% of the total number of papers published, which is far more than other countries.

Research related to periodontitis and aging has been conducted in 120 countries and regions. **Figures 2B, C** show the heat map and line graph of the annual publication volume of the top ten countries during the past 20 years. The top five countries with the highest publication volumes are the United States, Brazil, China, Japan and Germany. The percentage of papers published in the United States is 23.02%, far exceeding that of other countries.

The number of citations for papers published in the United States is 41,954 (**Supplementary Table S2**), significantly surpassing all other countries/regions. Additionally, the citation/publication ratio (40.97%) places the United States as the second highest globally. Although China has the third highest number of publications, its citation/publication ratio is only 17.26%, the lowest among the ten countries, which suggests that the quality of China’s publications is not high. Despite a significant difference in the number of publications compared to the United States, the citation/publication ratio for the United Kingdom (56.03%) ranks first globally. This indicates the superior quality of materials published from the UK. The network of cooperation is shown in **Figure 2D**, with close cooperation between the US, the largest producer, and Korea and Japan. The US also has cooperative relationships with countries such as China, Germany and the UK. From the heat map of paper publications, it can be observed that since 2002, the United States has published a greater number of articles. However, the number of articles published by China has surged in recent years and caught up with the United States by 2021. The United States not only has a significantly larger number of publications compared to other countries, but its centrality value also reached 0.46, indicating a leadership role in the development of the field. The remaining countries are still in the process of development in this field.

### 3.1 Institutions

3807 organizations systematically published articles related to periodontitis and aging. Among the top 10 institutions with the highest number of publications, six are from the United States, two from Switzerland, one from Finland and one from South Korea. Karolinska Instiv has published the most literature in this field (116 papers, 3,351 citations, 21.86 citations per paper). Univ Helsinki (105 papers, 3,315 citations, 57.56/paper) ranked second and Univ Washington (86 papers, 8,051 citations, 16.37/paper) ranked third (**Figure 4A**). It can be seen that institutions between countries prefer to cooperate with their own domestic institutions and there is a lack of international cooperation, so we call for the strengthening of cooperation between domestic and foreign institutions to break down academic barriers.

### 3.2 Journal

**Figure 3:**
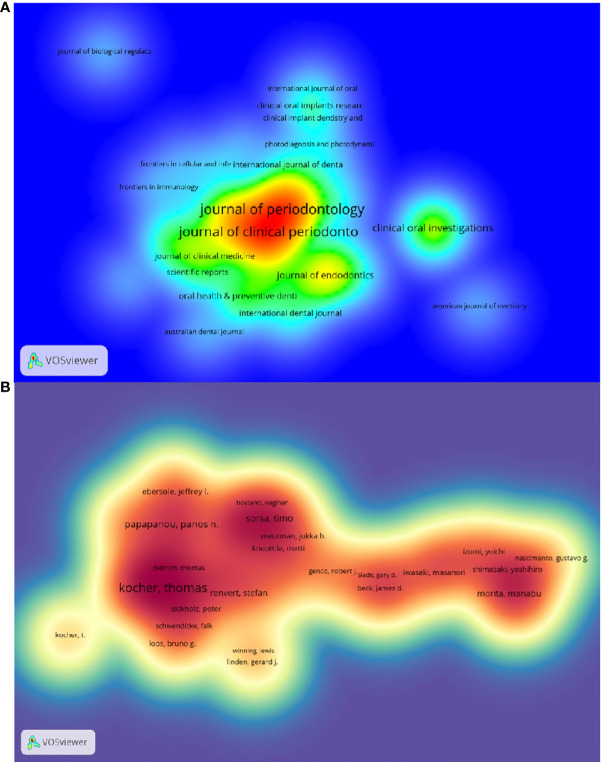
**(A)** Density map of journal postings. **(B)** Density map of author postings.

The top 10 journals with the highest number of publications are presented in **Supplementary Table S3** and **Figure 3A**. The *Journal of Periodontology* (522 articles, 11.74%) is the most published journal in this field. It was followed by *journal of clinical periodontology* (461 articles, 10.36%),*journal of periodontal research* (166 articles, 3.73%)和clinical oral investigations (138 articles, 3.10%). Among the 10 most prolific journals, journal of dental research had the highest IF of 7.6. 50% of the journals are categorized as Q1 and the remaining 50% are categorized as Q2.

**Figure 4:**
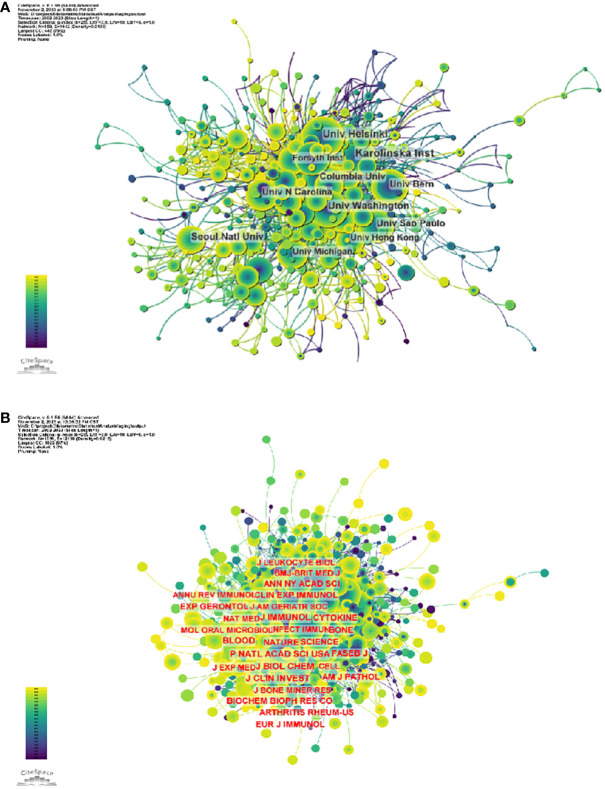
**(A)** Institutional Collaboration Network Diagram **(B)** Journal co-citation network diagram.

The impact factor of a journal is determined by the frequency with which it is cited and reflects the significant impact the journal has had on the scientific community. The most cited journal was J PERIODONTOL (3601), followed by J CLIN PERIODONTOL(3466)and J DENT RES (2711) (**Supplementary Table S5** and **Figure 4B**). Among the top 10 most co-cited journals, LANCET was cited 1062 times and had the highest impact factor (168.9). And 60% of the co-cited journals were first quarter journals.

The thematic distribution of scholarly publications is shown by a double map overlay (**Supplementary Figure S1**). The colored tracks indicate citation links, with the citing journal on the left and the cited journal on the right. After analyzing the data, we have identified three primary citation paths, each color-coded for clarity. (1) Research published in molecular/biology/immunology journals is predominantly cited by studies in molecular/biology/genetics and health/nursing/medicine journals. (2) Research reports in medicine/health/clinical research receive the most citations from journals in molecular/biological/genetics, health/nursing/medicine, and dermatology/dentistry/surgery fields. (3) Research reports in the dentistry/dental/surgery field are cited by journals primarily in the medical/immunology field.

The top 10 authors with the highest number of publications related to periodontitis and aging are shown in **Table S6** and **Figure 3B**. These authors have published a total of 276 papers, accounting for 6.21% of the total in the field. Kocher, Thomas (50 papers) published the most research papers, following papapanou, panos n. (31 papers) and holtfreter, birte (28 papers).

**Figure 5:**
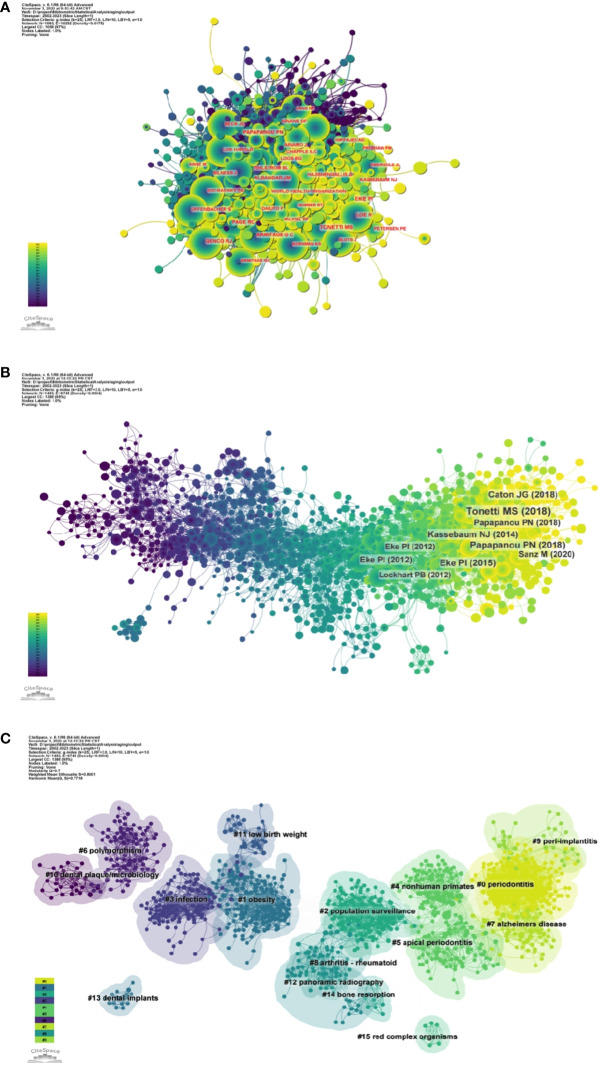
**(A)** Author co-citation network map. **(B)** Network diagram of co-cited literature. **(C)** Clustering map of co-cited literature.

**Figure 5A** and **Supplementary Table S1** respectively show the top 10 authors with the highest number of total citations and the highest number of total citations. 78 authors have a total of more than 100 citations, demonstrating the high reputation and impact of their research. The largest nodes are associated with the most co-cited authors, including TONETTI MS (698 citations), EKE PI (672 citations), and PAGE RC (534 citations).

### 3.3 Commonly cited references

Using a ten-year time slice with a time frame of 2013 to 2023, the co-cited reference network had 1453 nodes and 6745 links (**Figure 5B**). According to the top 10 most co-cited articles, the JOURNAL OF PERIODONTOLOGY (IF=6.7) entitled “Staging and grading of periodontitis: framework and proposal of a new classification and case definition” (12) was the most co-cited reference with Tonetti MS as the first author. We found that most of the 10 most co-cited articles were foundational literature related to periodontitis and aging.

**Figure 6:**
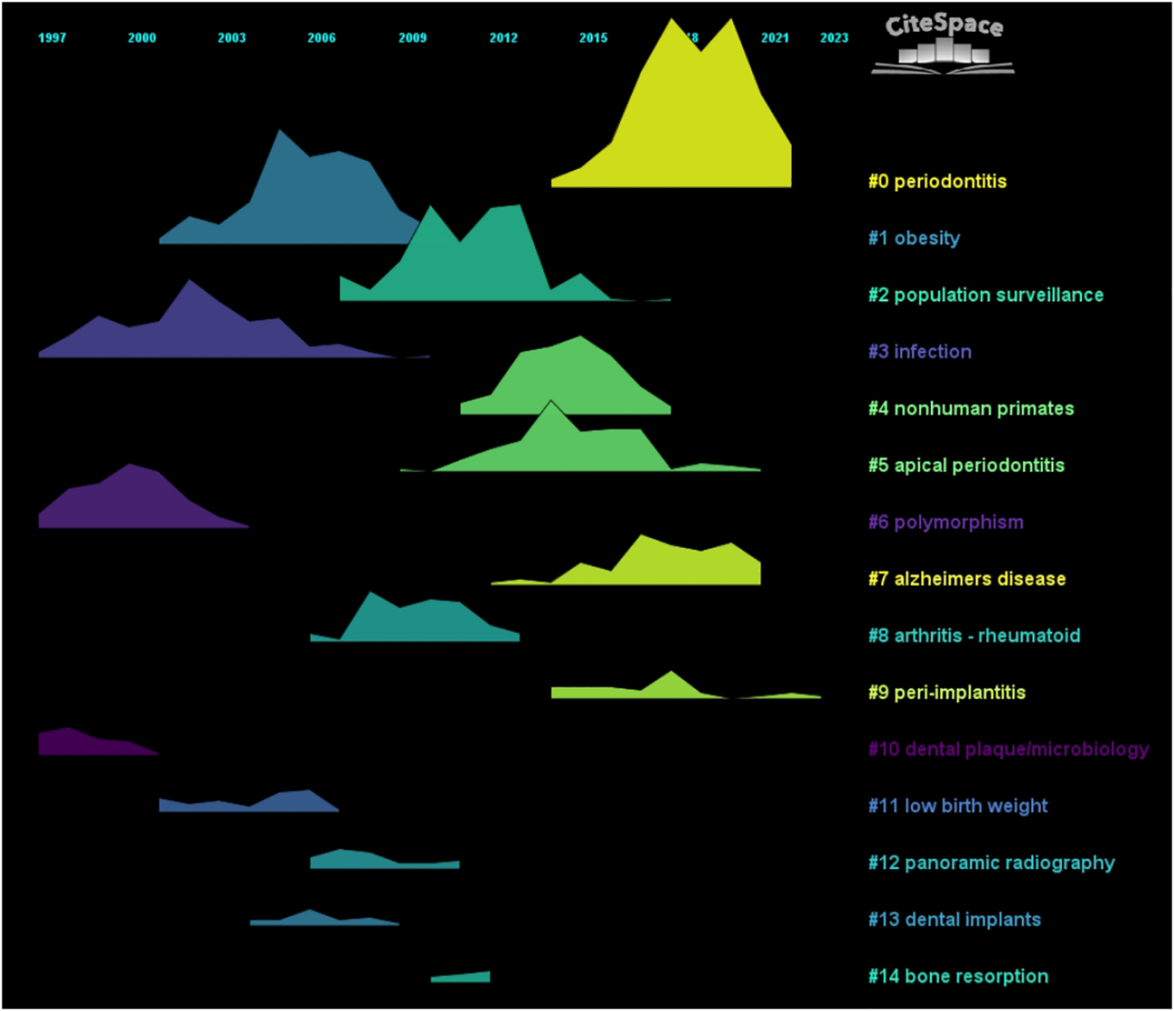
Volcano map of co-cited literature.

We performed co-citation reference clustering and timeline analysis (**Figures 5C**, **6**). We found that obesity (cluster1), infection (cluster3), polymorphism (cluster6), dental plaque/microbiology (cluster10), and low birth weight (cluster 11) were the population surveillance (cluster2), nonhuman primates (cluster4), apical periodontitis (cluster5), and arthritis-rheumatoid (cluster8), panoramic radiography (cluster12), dental implants (cluster13), and bone resorption (cluster14) are the hotspots of research in the mid-term, while periodontitis (cluster0), alzheimers Periodontitis (cluster0), alzheimers disease (cluster7), and peri-implantitis (cluster9) are the current hot topics and trends in this field.

### 3.4 Keyword analysis

**Figure 7:**
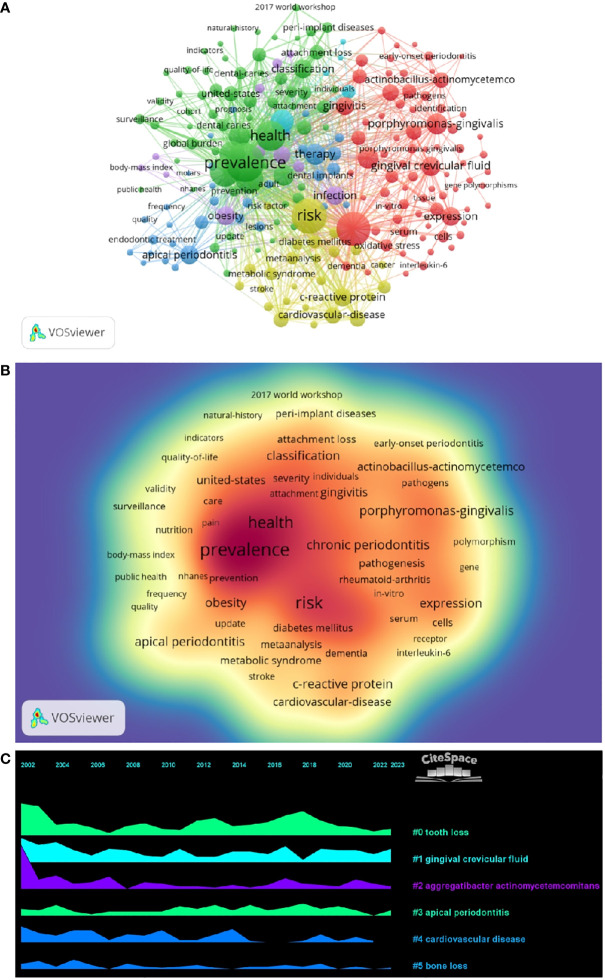
**(A)** Keyword clustering network diagram. **(B)** Keyword Density Map. **(C)** Keyword volcano map.

**Figure 8:**
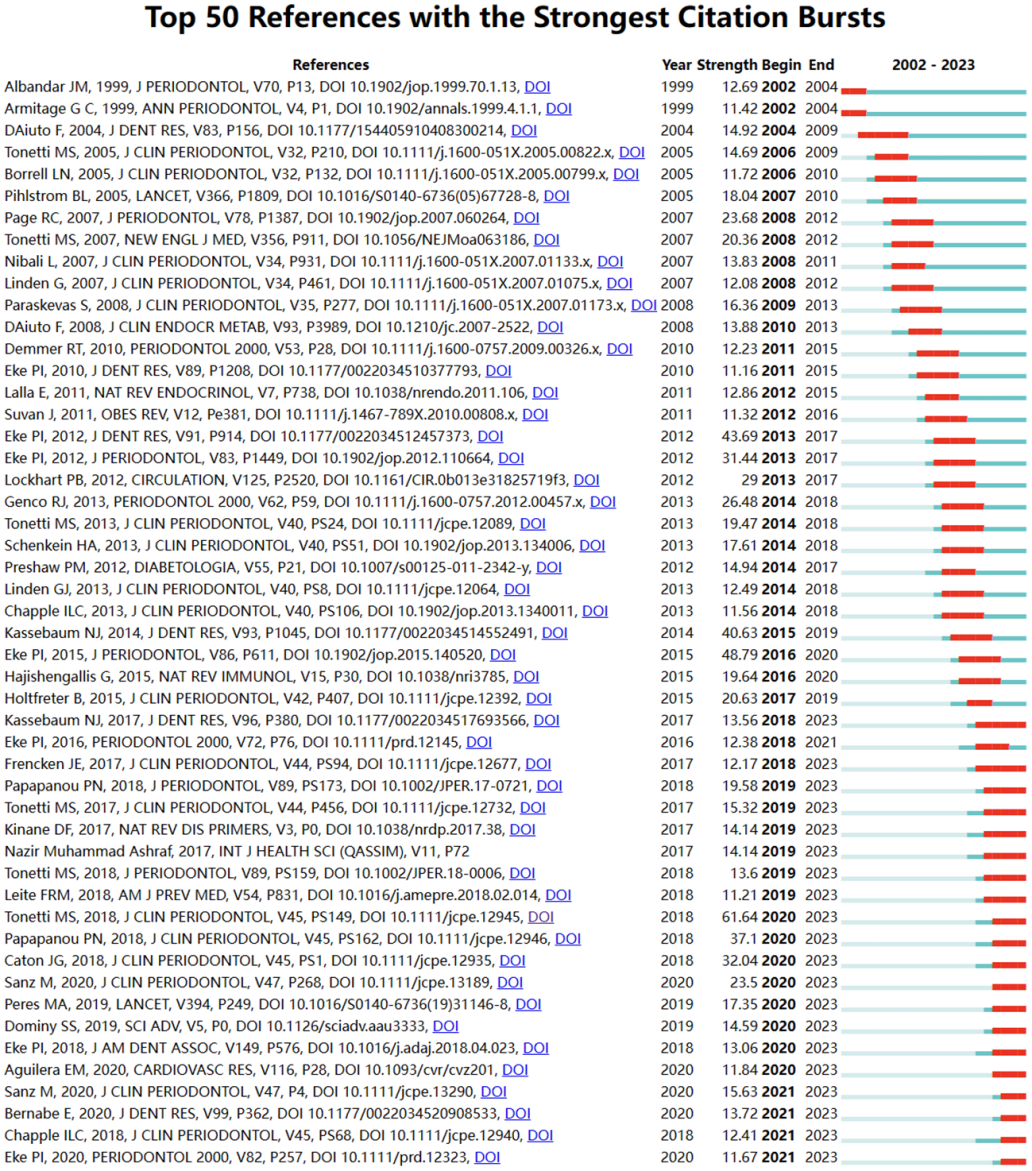
Co-cited literature emergence map.

By analyzing keywords, we can get a quick overview of a field and its direction. According to the co-occurrence of keywords in VOSwiever, the most popular keyword was PREVALENCE (747), followed by RISK (640), INFLAMMATION (600), and HEALTH (489) (**Table 1** and **Figures 7A, B**). We constructed a network containing 217 keywords with at least 30 occurrences (**Figure 8**), yielding a total of 6 different clusters. Cluster 1 (red) had 76 keywords including inflammation, biomarkers, chronic periodontitis, oxidative stress, peophyromonas-gingivalis, subgingival microbiota, bone loss, cytokines. group 2 (green) has 67 keywords, including prevalence, epidemiology, tooth loss, oral health, global burden, risk-factors, nutrition. group 3 contains 32 keywords (blue), including therapy, dental implants. Includes therapy, dental implants, alveolar bone loss, diagnosis, radiography, apical periodontitis, surgery, management Group 4 contains 26 keywords (in yellow) and includes risk, diabetes, metabolic syndrome, blood pressure, insulin-resistance, c-reactive protein, markers, glycemic control. group 5 contains 11 keywords (purple), including obesity, risk factors, weight, overweight, body-mass-index. group 6 contains 5 keywords (sky blue), including cigarette-smoking, smokers, tobacco smoking, and young-adults. a volcano map to visualize the research hotspots over time (**Figure 7C**).

**Table 1:** Table of high-frequency keywords.

Rank	Keyword	Counts	Rank	Keyword	Counts
1	prevalence	747	11	therapy	269
2	risk	640	12	porphyromonas-gingivalis	266
3	inflammation	600	13	chronic periodontitis	265
4	health	489	14	risk-factors	265
5	epidemiology	485	15	gingival crevicular fluid	234
6	tooth loss	435	16	apical periodontitis	228
7	population	376	17	c-reactive protein	226
8	oral-health	323	18	classification	224
9	smoking	319	19	infection	221
10	adults	294	20	obesity	220

### 3.5 Co-cited references and keyword highlighting

Through CiteSpace, we derived the 50 most reliable citation bursts related to the field of periodontitis and aging (**Figure 8**). One of them, “Staging and grading of periodontitis: framework and proposal of a new classification and case definition” by Maurizio S. Tonetti (13), the reference with the highest burst intensity (61.64). Forty-eight of the 50 references were published between 2002 and 2023, indicating that these papers have been cited frequently over the past 20 years and that research on periodontitis and aging will remain of interest in the future.

**Figure 9:**
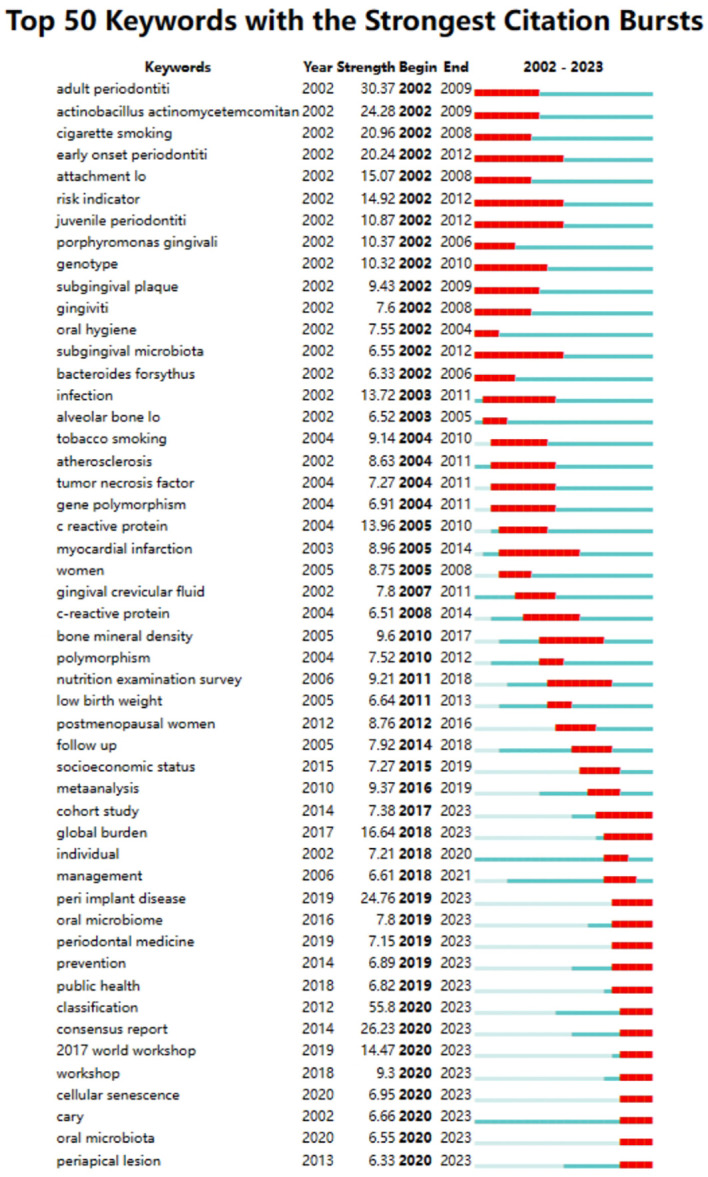
Keyword emergence map.

Among the 50 strongest burst keywords in the field, we focused on those that will still be mutating in 2023 (**Figure 9**), including “cohort study” (7.38 burst intensity), “global burden” (16.64 burst intensity), peri implant disease (24.76 burst intensity), oral microbiome (7.8 bursts), prevention (6.89 bursts), public health (6.82 bursts), classification (55.8 bursts), consensus report (26.23 bursts), 2017 world workshop (14.47 burst intensity), workshop (9.3 burst intensity), cellular senescence (6.95 burst intensity), cary (6.66 burst intensity), oral microbiota (6.55 burst intensity), and periapical lesion (6.33 burst intensity). (6.33 burst intensity).

## 4 Discussion

In this work, we assessed research trends and hot topics in the field of aging and periodontitis over the last 20 years through bibliometric analysis. We searched 2,339 articles and 901 reviews published from January 1, 2002, to October 25, 2023, respectively. During that research phase, only a few papers were published in the field prior to 2004. The number of papers has increased every year since 2005, rapidly increasing after 2017 and peaking in 2022, indicating that the field is growing rapidly and maintaining strong research interest. Therefore, we may speculate that research related to aging and periodontitis remains a hot topic for the future.

In the field of periodontitis and aging research, papers published in the United States were cited 41,954 times, far exceeding all other countries/regions, and it also had the second highest citation/number of papers ratio (40.97%). A Although China has the third largest number of papers in this field in the world, its citation/publication ratio is only 17.26%, indicating that the quality of Chinese publications still needs improvement. It is worth noting that although the UK has a low number of publications, its citation/publication ratio (56.03%) is among the highest in the world, indicating the high quality of the material it publishes. The collaboration network indicates close cooperation between the United States, South Korea, and Japan, with the highest number of published papers. According to the heat map, it can be seen that in recent years, the U.S. has had a higher volume of publications since 2002, while China has seen a surge in publications in recent years and is gradually catching up with the U.S. by 2021. The United States not only has a much higher number of publications than other countries but also has a centrality score of 0.46, indicating its leading position in this field. The rest of the countries are far less than the United States, indicating that these countries are still in the process of development in this field.

In terms of institutions, 3807 institutions systematically published articles related to periodontitis and aging. Among the 10 institutions with the highest number of publications, six are from the United States, two from Switzerland, one from Finland, and the other from South Korea. Karolinska Instiv had the most publications in this area (116 papers, 3351 citations, 21.86 citations per paper). Univ Helsinki (105 papers, 3315 citations 57.56 citations/paper) ranked second and Univ Washington (86 papers, 8051 citations, 16.37 citations/paper) ranked third. It can be seen that institutions between countries prefer to cooperate with their own domestic institutions, and there is a lack of international cooperation, so we call for the strengthening of cooperation between domestic and foreign institutions to break down academic barriers. Among all the authors who have published literature related to periodontitis and aging, **Table 2** and **Figure 5B** list the 10 authors who have published the most papers. The top 10 authors have collectively published 276 papers, accounting for 6.21% of all papers in this field. kocher, thomas (50 papers) published the most research papers, following by papapanou, panos n. (31 papers) and holtfreter, birte (28 papers). According to the top 10 most co-cited articles (**Table 3**), the JOURNAL OF PERIODONTOLOGY (IF=6.7) entitled “Staging and grading of periodontitis: framework and proposal of a new classification and case definition” (18) was the most co-cited reference, demonstrating the importance of this journal among periodontal journals, with Tonetti MS as the first author, demonstrating his centrality to the field. We found that most of the 10 most co-cited articles were foundational literature related to periodontitis and aging.

**Table 2:** Top 10 literature co-citation table.

Rank	Title	Journal IF(2021)	Author(s)	Total citations
1	Staging and grading of periodontitis: Framework and proposal of a new classification and case definition	JOURNAL OF CLINICAL PERIODONTOLOGY(IF=6.7)	Tonetti, Maurizio S.	239
2	Periodontitis: Consensus report of workgroup 2 of the 2017 World Workshop on the Classification of Periodontal and Peri-Implant Diseases and Conditions	JOURNAL OF CLINICAL PERIODONTOLOGY(IF=6.7)	Tonetti, Maurizio S.	148
3	A new classification scheme for periodontal and peri-implant diseases and conditions - Introduction and key changes from the 1999 classification	JOURNAL OF CLINICAL PERIODONTOLOGY(IF=6.7)	Tonetti, Maurizio S.	125
4	Update on Prevalence of Periodontitis in Adults in the United States: NHANES 2009 to 2012	JOURNAL OF PERIODONTOLOGY(IF=4.3)	Eke, Paul I.	123
5	Global Burden of Severe Periodontitis in 1990-2010: A Systematic Review and Metaregression	JOURNAL OF DENTAL RESEARCH(IF=7.6)	Kassebaum, N. J.	91
6	Prevalence of Periodontitis in Adults in the United States: 2009 and 2010	JOURNAL OF DENTAL RESEARCH(IF=7.6)	Forbes, Josephine M	90
7	Periodontitis: Consensus report of workgroup 2 of the 2017 World Workshop on the Classification of Periodontal and Peri-Implant Diseases and Conditions	JOURNAL OF PERIODONTOLOGY(IF=4.3)	Tonetti, Maurizio S.	80
8	Periodontitis and cardiovascular diseases: Consensus report	JOURNAL OF CLINICAL PERIODONTOLOGY(IF=6.7)	Sanz, Mariano	77
9	Update of the Case Definitions for Population-Based Surveillance of Periodontitis	JOURNAL OF PERIODONTOLOGY(IF=4.3)	Eke, Paul I.	65
10	Periodontal Disease and Atherosclerotic Vascular Disease: Does the Evidence Support an Independent Association? A Scientific Statement From the American Heart Association	CIRCULATION (IF=37.8)	Lockhart, Peter B	60

**Table 3:** Reported TLR9 -related content.

Drug(optimal dose)	Target	conclusion	References
Erythromycin (DPG3:10μg/ml) Tetracycline (MF1:1 μg/ml)	TLR2, -3, -4, -6, and -9	1.Sensitization of Human Aortic Endothelial Cells to Lipopolysaccharide via Regulation of Toll-Like Receptor 4 by Bacterial Fimbria-Dependent Invasion 2.invasive P. gingivalis infection of primary aortic endothelial cells results in increased TLR expression on the cell surface. 3.priming of endothelial cells by invasive P. gingivalis infection leads to increased binding of PAMPs and the induction of TLR-dependent inflammatory responses	Yumto H, et al, 2005 (69)
P. gingivalis LPS (HPLCs:10 mg/ml)	TLR2 and -4	Gram-negative periodontal bacteria or their LPS might play a role in triggering TLR2 and/or TLR4, and be of importance for the immune responses in periodontitis	Sun Y, et al, 2010 (70)
Gingivalis (HGFs)	TLR2,-4 and -9	P. gingivalis infection induces TLR2 and TLR9 upregulation in patients with CP. P. gingivalis–induced TLR2 expression in HGFs is partially dependent on TNF-a and may lead to sensitization of HGFs to bacterial components encountered in the periodontal microenvironment	Wara-aswapati N, et al, 2013 (71)
P. gingivalis LPS (HPDLFs:10mg/L)	TLR2,-4 and MAPK	In hPDLFs, P. gingivalis LPS suppresses bone sialoprotein and enhances IL-8 gene and protein expression via TLR2 and ERK1/2 or the p38 MAPK signaling pathway, respectively.	Zhang Y, et al, 2015 (72)
	TLR2,-4,-5,-7,-9 and IFN-α1	The expression levels of TLR2, -4, -7, and -9 were significantly higher in periodontitis lesions than gingivitis lesions. The expression level of TLR5 was comparable to levels of TLR2 and -4; however, no significant difference was found between gingivitis and periodontitis. Although the expression of IFN-a1 mRNA was higher in periodontitis lesions compared with gingivitis lesions, the level was quite low. Only a few pDCs were found in some periodontitis specimens. No difference was found for antibody-positivity between gingivitis and periodontitis.	Kajita K, et al, 2007 (73)
live P. gingivalis (2x109 CFU)	TLR9	1.TLR9-mediated inflammation promotes P. gingivalis-induced periodontal bone loss. 2.Lack of TLR9 signaling suppresses inflammation in gingival tissues of P. gingivalis-infected mice. 3.Lack of TLR9 signaling leads to decreased cytokine production in splenocytes and macrophages challenged with P. gingivalis. 4.Lack of TLR9 signaling affects cytokine production in splenocytes and macrophages challenged with TLR2 and TLR4 agonists.	Kim PD, et al, 2015 (74)
	TLR9	The TLR9 haplotype has a protective effect against the development of CP and, in contrast, haplotype can increase the susceptibility of this disease in the Czech population.	Holla LI, et al, 2010 (75)
	TLR-9,AIM2 and DAI	This study is the first report of AIM2 and DAI receptor expression in periodontal tissues and further confirms increased TLR-9 expression, as well as reporting enhanced TLR-8 expression at CP sites.	Sahingur SE, et al. 2013 (76)
	TLR9	Expression of cytokeratin 19 (CK19) was markedly increased in the basement membranes of the oral epithelium and in all layers of the pocket epithelium where it caused evident cell proliferation and migration of sulcular epithelial cells into the lamina propria of periodontitis tissue. TLR4 and the cytoplasmic NLRP3 were expressed in all sections examined regardless of disease state. However, expression of TLR9-,CK19-and collagenolytic matrix metalloproteinase-13 and activated NF-kB subunit p65 was more commonly found in periodontitis tissues than in gingivitis tissues.	Chen YC, et al,2012 (77)
	TLR4, NOD1 and NOD2	Using human peripheral blood monocytes (HPBM) And murine bone-marrow-derived macrophages (BMDM) from wild-type (WT) and Toll-like receptor (TLR)-specific and MyD88 knockouts (KOs), we demonstrated that heat-killed Campylobacter concisus, Campylobacter rectus, Selenomonas infelix, Porphyromonas endodontalis, Porphyromonas gingivalis, and Tannerella forsythia mediate high immunostimulatory activity. Campylobacter concisus, C. rectus, and S. infelix exhibited robust TLR4 stimulatory activity. Studies using mesothelial cells from WT and NOD1-specific KOs and NOD2-expressing human embryonic kidney cells demonstrated that Eubacterium saphenum, Eubacterium nodatum and Filifactor alocis exhibit robust NOD1 stimulatory activity, and that Porphyromonas endodontalis and Parvimonas micra have the highest NOD2 stimulatory activity. T	Marchesan J, et al, 2016 (78)
OMVs (HEK-Blue cells)	TLR2,-4,-7,-8,-9,NOD1 and NOD2	P. gingivalis OMVs induced strong TLR2 and TLR4-specific responses and moderate responses in TLR7, TLR8, TLR9, NOD1 and NOD2 expressing-HEK-Blue cells. Responses to T. forsythia OMVs were less than those of P. gingivalis and T. denticola OMVs induced only weak responses.	Cecil JD, et al, 2016 (79)
	TLR1,-2 and -4	Semiquantitative polymerase chain reaction and immunohistochemical analysis demonstrated that of 9 TLRs, the expression of TLR1, TLR2, and TLR4 was markedly enhanced in human atherosclerotic plaques. A considerable proportion of TLR-expressing cells were also activated, as shown by the nuclear translocation of nuclear factor-kB	Edfeldt K, et al, 2002 (80)
	TLR9	CpG DNA binds directly to TLR9 in ligand-binding studies. CpG DNA moves into early endosomes and is subsequently transported to a tubular lysosomal compartment. Concurrent with the movement of CpG DNA in cells, TLR9 redistributes from the ER to CpG DNA–containing structures, which also accumulate MyD88. Our data indicate a previously unknown mechanism of cellular activation involving the recruitment of TLR9 from the ER to sites of CpG DNA uptake, where signal transduction is initiated.	Latz E, et al, 2004 (81)
	TLR2,-4 and-9	TLR9 activation by F. nucleatum and TLR2 activation by both bacteria appear to be involved in HIV-1 reactivation; however, TLR4 activation had no effect.	González OA, et al, 2010 (82)

In bibliometric analyses, keyword bursts usually reflect hot topics in the research field (83), and keyword volcano maps can show the evolution of new research hotspots (84, 85). We found that obesity, infection, polymorphism, dental plaque/microbiology, and low birth weight were early research hotspots, population surveillance, nonhuman primates, apical periodontitis, arthritis-rheumatoid, panoramic radiography, dental implants and bone resorption are mid-term hotspots. While periodontitis, alzheimers disease and peri-implantitis are current hot topics and trends in the field. In periodontitis, a large accumulation of microorganisms leads to the recruitment of polymorphonuclear neutrophils to the lesion site within the periodontal pocket. As the first line of defense against pathogens, the recruitment of neutrophils is influenced by various factors, including immune cell cytokines and chemotactic factors (86, 87). Early keyword hotspots reflect the exploration of periodontitis and the mechanisms of aging, and current keywords in the field reflect the aging phenomenon in which the possible link between disease and illness in the aging process and how to prevent and treat it will become a hotspot.

The analysis of co-occurring keywords indicates a close relationship between periodontitis and aging, consistent with previous research findings in recent years (88–90). Aging may trigger a decline in both innate and adaptive immune functions in the body, as well as alterations in the functional efficacy of effector biomolecules, leading to immune aging, immune activation, and inflammatory processes. This may be one of the plausible mechanisms by which aging may lead to increased susceptibility to periodontitis (91). Notably, the *in vivo* microenvironment associated with aging may additionally increase the complexity of defects in innate immunity and adaptive function (92–94). In addition, the close relationship between aging and bone metabolism was analyzed by keywords. Many immune and non-immune cells in periodontal tissue interact with osteoblasts and osteoclasts, which can regulate the balance of physiological bone formation and bone resorption (95, 96). Therefore, bone metabolism is regulated by various factors, including aging. In particular, aging disrupts the balance between bone remodeling and metabolism, leading to increased bone resorption, changes in bone structure, and decreased fracture resistance (16).

Based on results from keyword co-occurrence analysis, we also identified several terms related to systemic diseases, such as Alzheimer’s disease, atherosclerosis, and rheumatoid arthritis. These findings are consistent with recent research trends regarding the association between periodontitis and systemic diseases. These complex, multi-factorial diseases share common characteristics with periodontitis, including accelerated aging (97–99).

In recent years, Hajishengallis found that periodontitis can lead to dysbiosis, which is a serious risk to human health. In addition, localized treatment of periodontitis may serve as an additional indicator for the reduction of systemic inflammation and concomitant diseases (100, 101). There is substantial evidence suggesting that periodontitis may be influenced by systemic low-grade inflammation (102). Generally, periodontitis shows a bidirectional relationship with systemic inflammatory diseases. Aging is considered as a common factor that may explain the pathophysiological mechanisms underlying various inflammatory diseases (103–105). Therefore, aging may be a critical therapeutic target for inflammatory diseases.

Furthermore, citespace’s keyword exploration also helps to reveal future trends. We conclude that macrophage imbalance, oxidative stress may be current research hotspots. Meanwhile, pyroptosis and TLR9 may be potential research directions and targets.

### 4.1 Macrophage imbalance

Macrophages, as part of the innate immune system, possess high plasticity. When exposed to local stimuli, macrophages can exhibit various active states, which are classified into 2 major subsets, depending on the stimuli: classically activated (M1) and alternatively activated (M2) (106–108). Dysfunction of macrophages has been recognized as a key factor in periodontitis. Understanding the roles of different macrophage phenotypes contributes to the comprehension of potential mechanisms underlying periodontitis and aids in the development of new therapeutic strategies. Pro-inflammatory M1-like macrophages and reparative M2-like macrophages play an important role in inflammation and tissue homeostasis in periodontitis. Periodontal pathogens can induce the differentiation of macrophages into M1 type by stimulating CD14, Toll-like receptors (TLRs), and NOD-like receptors (NLRs) on the surface of macrophages with LPS. M2 macrophages, on the other hand, exert anti-inflammatory effects and play a role in wound healing and tissue repair. In the early stages of periodontitis, M1 macrophages dominate the infiltration of periodontal tissues, and the proportion of M1 is positively correlated with the progression of periodontal inflammation. There is evidence suggesting an increase in the abundance of M1 macrophages and a decrease in M2 counts in periodontal tissues from patients with periodontitis compared to healthy controls (109–112). During the progression of periodontitis, on one hand, periodontal pathogens may induce an excessive inflammatory response by modulating metabolic pathways to skew macrophage polarization towards M1, while on the other hand, sites of alveolar bone destruction accumulate large numbers of M1 macrophages, leading to the production of IL-1β and TNF-α, upregulation of RANKL expression, and increased bone resorption (113, 114). However, different studies have shown that no alterations in the M1/M2 ratio in periodontal tissues from patients with periodontitis (115, 116). Furthermore, the quantity of M1 macrophages notably decreases during the periodontitis stage. Therefore, in the pathogenesis of periodontitis, it is likely that the roles of M1 pro-inflammatory macrophages and M2 anti-inflammatory macrophages are not as straightforward as previously thought. They exist in a dynamic equilibrium, and disruption of this balance can lead to persistent inflammation (117, 118). During the natural healing process of periodontitis, M1 macrophages are capable of phagocytosing microorganisms and matrix debris. They exhibit a high antigen-presenting capacity during the early stages of healing. Consequently, the number of M1 macrophages increases in the early healing stages and then rapidly declines thereafter. M2 macrophages, on the other hand, peak in numbers during the later stages of healing (118–120). By producing IL-10 and reducing the expression of IL-6, M2 macrophages regulate the functions of Th17 and Treg cells, thereby influencing their anti-inflammatory and reparative properties (121, 122). In addition, the polarization of macrophages also undergoes changes with age. Research indicates that while there is no significant difference in the quantity of macrophages between young and old mice, the expression of M1-related markers, pro-inflammatory cytokines, and chemokines significantly increases in aged mice.[ (112, 123) Therefore, the aging immune system may play a crucial role in alveolar bone loss (124). Interventions targeting the aging immune system could emerge as a novel approach to managing periodontitis in elderly patients. Currently, the standard treatment for periodontitis typically involves mechanical removal of disrupted biofilms to control inflammation (125). However, this approach doesn’t always successfully halt the progression of the disease. Hence, exploring additional therapeutic targets contributes to the advancement of periodontal treatment. While therapeutic modulation of macrophage phenotypes has not yet been utilized in the treatment of periodontitis patients, inhibiting macrophage polarization to the M1 type or increasing the numbers and ratio of M2 macrophages may alleviate the progression of periodontitis. Therefore, the transition of macrophage phenotypes could potentially serve as a promising therapeutic target for periodontitis treatment, holding significant promise (**Table 4**).

**Table 4:** Reported macrophage polarization-related content.

Drug(optimal dose)	Target	Conclusion	References
	P53	The activation of p53 gene could alleviate periodontitis by reducing M1-type macrophage polarization	Liu, T et al.2024 (27)
Dimethyloxalylglycine (C57BL/6 male mice:1.25 mg/kg)	HIF-1α	Dimethyloxalylglycine Inhibits M1-like Polarization of RAW264.7 Macrophages and Mouse Bone Marrow Macrophages (BMM) for the Treatment of Periodontitis	Chen, Mei-Hua et al.2021 (28)
Leptin (C57BL/6 male mice:40 ug/mouse,Qd, ip)	NLRP3	Leptin aggravates the periodontal response to the ligature by promoting M1 macrophage polarization via the NLRP3 inflammasome	Han, Y et al.2022 (29)
	UCP2	UCP2 controls M1 macrophage activation by modulating reactive oxygen species (ROS) production	Yan, X et al.2020 (30)
	A20	A20 inhibits periodontal bone resorption and NLRP3-mediated M1 macrophage polarization	Hou, Liguang et al.2020 (31)
	miR-143-3p	Inflammatory PDLSCs facilitate M1 macrophage polarization through the exosomal miR-143-3p-mediated regulation of PI3K/AKT/NF-κB signaling, providing a potential new target for periodontitis treatment.	Wang, Yazheng et al.2023 (32)
M2-exos male SD rats		M2‐exos drive an appropriate and timely macrophage reprogramming from M1 to M2 type, which resolves chronic inflammation and accelerated periodontal healing.	Cui, Y et al.2023 (33)
Exo-TNF-α (C57BL/6NCrSlc female:20 μ g/mouse,Qd)		TNF-α stimulation not only increased the amount of exosome secreted from GMSCs, but also enhanced the exosomal expression of CD73, thereby inducing anti-inflammatory M2 macrophage polarization	Nakao, Y et al.2020 (34)
Mo-BGC (C57BL/6 mice:0.1μmol/mL)		Induced M2 polarization by enhancing the mitochondrial function of macrophages and promoted a cell metabolic shift from glycolysis toward mitochondrial oxidative phosphorylation.	He, X.T et al.2022 (35)
PDLSCs		PDLSCs were able to induce M2 macrophage polarization instead of M1 polarization, and capable of enhancing M2 macrophage polarization induced by IL-4 and IL-13.	Liu, J et al.2022 (36)
M2-Exo (C57BL/6mice:30ul,100 μg/ml)	IL-10/IL-10R	The reparative M2-like macrophages could promote osteogenesis while inhibiting osteoclastogenesis *in vitro* as well as protect alveolar bone against resorption *in vivo* significantly.	Chen, Xutao et al.2022 (37)
Sulforaphene (SFE) (male C57BL/6 mice:20mg/kg, Qd, ip)	DCIR	SFE effectively inhibits M1 polarization while promoting M2 polarization, ultimately suppressing periodontitis.	Liao, Y et al.2023 (38)
G3@SeHANs (C57BL/6 male mice:30ul, 1 mg/mL)		G3@SeHANs regulates the mononuclear phagocyte system in the periodontitis environment and promotes M2 macrophage phenotype over M1 macrophage phenotype	Huang, H et al.2024 (39)
Interleukin-37 (RAW264.7 cells:50μL,20μg/mL)	NLRP3	IL-37 prevents the progression of periodontitis by suppressing NLRP3 inflammasome activation and mediating M1/M2 macrophage polarization.	Yang, L et al.2024 (40)
Glipizide (C57BL/6 male mice:10 mg/kg, Qd, ig)	.	Glipizide inhibited the LPS-induced migration of BMMs but promoted M2/M1 macrophage ratio in LPS-induced BMMs via activation of PI3K/AKT signaling	Guo, Xet al.2023 (41)
Quercetin-Loaded Ceria Nanocomposite Potentiate Dual-Directional Immunoregulation (Rat:50 µg mL -1)		Such nanocomposite can control the phenotypic switch of macrophages by not only inhibition of M1 polarization for suppressing the damage in the destructive phase but also promotion of M2 polarization for regenerating the surrounding tissues in reparative phase of periodontal disease.	Wang, Y et al.2021 (42)
CMC2.24 (Rat Mφ:5 µM)		CMC2.24 appears to be a potent inhibitor of the pro-inflammatory M1 phenotype; and a promotor of the pro-resolving M2 phenotype, thus acting like a crucial “switch” to reduce inflammation.	Deng, J et al.2023 (43)
	RGS12	Knockdown of RGS12 in macrophages promotes macrophage reprogramming to M2 type, and macrophage migration in response to lipopolysaccharide stimulation	Yuan, G et al.2022 (44)
BMSC-sEVs (male SD rats:500 μg/mL)		Promoting periodontal regeneration by modulating transforming growth factor-β1 (TGF-β1) expression and the ratio of type 2 macrophages to type 1 macrophages (M2/M1)	Liu, L et al.2021 (45)

### 4.2 Oxidative stress

Oxidative stress is an integral part of the pathogenesis of periodontitis and is the result of an imbalance between the oxidative and antioxidant systems in the body, an over-oxidized state caused by an overproduction of ROS and/or a lack of antioxidant defenses (126). A causal relationship between ROS-mediated oxidative stress and periodontal disease has been demonstrated (127, 128). Studies have shown that the serum levels of superoxide are significantly higher in periodontitis patients, especially in chronic periodontitis, than in healthy individuals. In addition, the levels of malondialdehyde (MDA), a biomarker of lipid peroxidation, and 8-hydroxydeoxyguanosine (8-OHdG), a marker of oxidative DNA damage, were significantly elevated in saliva and gingival sulcus of patients with chronic periodontitis compared with healthy periodontal tissues (3, 129). Oxidative stress induced by the antimicrobial response during periodontitis may be an important cause of tissue damage. ROS play an important role in the pathomechanism of periodontitis.Gram-negative anaerobes colonizing dental plaque trigger the recruitment and activation of neutrophils, which subsequently produce a range of antimicrobial factors during phagocytosis of periodontal pathogens and release an excess of ROS through the NADPH oxidase pathway (130, 131). During phagocytosis, free radicals are the end-products of functional roles played by the mitochondria of polymorphonuclear neutrophils, mainly through lipid peroxidation (132–134). This leads to an oxidative imbalance that triggers a pro-inflammatory mechanism, which in turn promotes osteoclastogenesis, thus leading to alveolar bone resorption in patients with periodontitis (127, 135–139). In addition, reactive oxygen species affect Nuclear factor-erythroid 2-related factor 2 (Nrf2),Nrf2 down-regulation is associated with the progression of periodontitis, and finally, through direct damage to extracellular connective tissues (in addition to the bone itself), the production of reactive oxygen species is responsible for the loss of attachment that leads to periodontal destruction (140, 141).At the same time, reactive oxygen species production activates the NF-κB signaling pathway, which mediates the phosphorylation and degradation of NF-κB inhibitor a of NF-κB (IκBα) and the nuclear translocation of P65, a subunit of NF-κB, which promotes proinflammatory cytokines (such as interleukin-1, interleu-kin-6, and tumor necrosis factor-alpha), which can act directly or indirectly on periodontal tissues to cause bone resorption (142–144).In addition, increased reactive oxygen species disrupt the homeostatic relationship between bone formation and resorption via the RANKL/osteopro-tegerin axis, and thus oxidative stress-mediated inflammation may be one of the pathways leading to the development of periodontal disease. This means that conventional prophylaxis and treatment focusing on bacterial infection-mediated periodontal disease seems to be insufficient, especially when initial periodontal treatment fails to alleviate inflammation, and antioxidant scavenging of ROS to reduce overburdened inflammation is considered to be an effective way to stop the progression of periodontitis (**Table 5**).

**Table 5:** Reported oxidative stress-related content.

Drug(optimal dose)	Target	conclusion	References
Silibinin (Rat:150 mg/kg)	NrF2	Silibinin exhibits anti-inflammatory and antioxidant properties against periodontitis by upregulating Nrf2 expression	Li, X et al., 2023 (46)
Curcumin (hPDLSCs:0.1 μM)	NrF2	Curcumin could promote the osteogenesis of hPDLSCs, and the effect is related to the PI3K/AKT/Nrf2 signaling pathway.	Xiong, Y.et al.2020 (47)
Resveratrol (Rat:5mg/kg Hgfs:50μM)	NrF2	Resveratrol alone augmented HO-1 induction via Nrf2-mediated signaling.	Bhattarai, G.et al.2016 (48)
Metformin (hPDLSCs:100μM)	NrF2	Metformin activates the Nrf2 signaling pathway in PDLSCs, which not only promotes osteogenic differentiation of PDLSCs, but also protects PDLSCs from oxidative stress-induced injury	Jia, L.et al.2020 (49)
Quercetin (hPDLSCs:5 μM)	NrF2	quercetin activated NRF2 signaling in the periodontal ligaments, reduced the OS level of mice with periodontitis, and slowed the absorption of alveolar bone *in vivo*.	Wei, Y. et al.2021 (50)
Four-Octyl itaconate (C57BL/6 male mice:50 mg/kg)	NrF2	Four-Octyl itaconate attenuates inflammation and oxidative stress via disassociation of KEAP1-Nrf2 and activation of Nrf2 signaling cascade.	Xin, L et al.2022 (51)
N-Acetyl-l-cysteine-Derived Carbonized Polymer Dots (C57BL/6 male mice:100mg/kg)	NrF2	N-Acetyl-l-cysteine-Derived Carbonized Polymer Dots may regulate redox homeostasis and promote bone formation in the periodontitis microenvironment by modulating the kelch-like ECH-associated protein l (Keap1)/nuclear factor erythroid 2-related factor 2 (Nrf2) pathway.	Liu, X et al.2023 (52)
Paeonol (Rat:80 mg/kg)	NrF2	paeonol protected against periodontitis-aggravated osteoclastogenesis and alveolar bone lesion via regulating Nrf2/NF-κB/NFATc1 signaling pathway.	Li, Y et al.2019 (53)
Baicalein (Rat:200mg/kg)	NrF2	Baicalein attenuates alveolar bone loss by upregulating NRF2	Zhu, C et al.2020 (54)
Chlorogenic acid (Hgfs:40μM)	NrF2	Chlorogenic acid attenuates inflammation in human gingival fibroblasts, possibly through CysLT1R/Nrf2/NLRP3 signaling.	Huang, X et al.2022 (55)
Notopterol (C57BL/6 male mice:20 mg/kg)	NrF2	Notopterol alleviates periodontal inflammation by activating the NRF2 signaling pathway	Zhou, J et al.2023 (56)
Epigallocatechin-3-gallate (Rat:200mg/kg)	NrF2	Epigallocatechin-3-gallate inhibits oxidative stress and inflammatory responses in the periodontitis model by modulating the Nrf2/HO-1/NLRP3/NF-κB p65 signaling pathway	Fan, Q et al.2023 (57)

### 4.3 Pyroptosis

Pyroptosis, a pro-inflammatory programmed cell death dependent on the Gasdermin family of proteins, plays an important role in the regulation of peridontal environmental homeostasis (145). Compared to apoptosis, pyroptosis occurs more rapidly as the cell swells until the membrane ruptures, releasing cellular contents and activating a strong inflammatory response (146, 147). Inflammatory mediators can cause collagen degradation and bone matrix resorption (148), causing destruction of periodontal hard and soft tissues. The initiation of cellular pyroptosis is dependent on the activation of intracellular inflammatory vesicles and their downstream cysteinyl aspartate-specific proteases1 (caspase-1), as well as the production of active fragments of the key pyroptosis protein Gasdermin. The invasion of periodontal pathogens induces an inflammatory response in the host, the molecular mechanism of which involves the activation of multiple inflammatory vesicles and triggers cellular pyroptosis, as well as the release of a large number of inflammatory factors, such as interleukin (IL)-1β, IL-18, and others, which mediate the destruction of periodontal tissues (149). The inflammatory response of periodontopathogenic bacteria in the periodontium is characterized by the release of a wide range of inflammatory factors. There is growing evidence that focal death plays a role in periodontitis pathology, such as Porphyromonas gingivalis, which can induce an inflammatory response through focal death. CASP4/GSDMD triggered by bacterial LPS leads to focal death of periodontal ligament stem cells in periodontitis patients (150). In the early stages of inflammation, gingival epithelial cell pyroptosis disrupts the epithelial barrier by interfering with intercellular junctions. When inflammation progresses, cell pyroptosis can further cause gingival fibroblast death, impaired migration of periodontal fibroblasts, impaired migration of osteoblasts, and active osteoclasts, which will be macroscopically manifested as loss of attachment and alveolar bone resorption. At the same time, the cellular contents released by pyroptosis, such as inflammatory factors and mtROS, can stimulate the production of MMP, causing collagen degradation in gingival and periodontal tissues. In summary elevated levels of focal death in periodontitis promote the secretion of active inflammatory factors (IL-1β, IL-18), which amplifies the inflammatory response and leads to an overactive immune response; this ultimately reduces bone formation, enhances bone resorption by up-regulating RANKL, exacerbates the destruction of periodontal tissues, and inhibits their regeneration. However, the regulation of cellular pyroptosis related to the osteoclastic mechanism needs to be further explored (**Table 6**).

**Table 6:** Reported pyroptosis -related content.

Drug(optimal dose)	Target	conclusion	References
MARK4 inhibitors (OTSSP167 and Compound 50) and small interference RNA )	MARK4	Inhibition of MARK4 decreased LDH release, IL-1β and IL-18 production, ASC speck formation, and the pyroptosis-related genes transcription.	Wang, L et al. (58)
Azgp1 knockout mice	NLRP3/caspase-1	AZGP1 participates in the pathogenesis of periodontitis by aggravating macrophage M1 polarization and pyroptosis through the NLRP3/caspase-1 pathway.signaling pathway.	Yang, S et al. (59)
Eldecalcitol (Rat:5mg/kg Hgfs:5nM)	NLRP3	ED-71 inhibits cellular pyroptosis by decreasing the activation of NLRP3 inflammatory vesicles.	Huang, C et al. (60)
z-YVAD-FMK(hPDLSCs:2 mM)	caspase-1	The NLRP1 inflammasome led to the activation of caspase-1 and the subsequent activation and releasing of IL-1β, and initiated pyroptosis.	Zhao, D et al. (61)
Construction of DEC2 overexpression vector (RAW 264.7)	caspase-11	Dec2 overexpression reduces levels of IL-1β and sequentially regulates caspase-11, thereby inhibiting pyroptosis.	He, D et al. (62)
Kynurenic (RAW 264.7:100uM)	Caspase1/NLRP3	Kynurenic significantly suppressed macrophage pyroptosis induced by LPS.	Gao, Y et al. (63)
Construction of TET1 overexpression vector (OCCM-30)	caspase-1	TET1 prevents the onset of cellular pyroptosis by inhibiting caspase-1 activation.	Peng, Y et al. (64)
Isoliquiritigenin (Hgfs:5μM)	NLRP3	Isoliquiritigenin attenuates Porphyromonas gingivalis-induced pyroptosis by inhibiting NLRP3 activation.	Lv, X et al. (65)
N-acetylcysteine(hPDLSCs:10mM)	NF-κB/Caspase-1	N-acetylcysteine reduces cellular pyroptosis by inhibiting the NF-κB/Caspase-1 signaling pathway.	Chu, Y et al. (66)
Metformin (mice:200mg/kg)	NEK7/NLRP3	Metformin reduces cellular pyroptosis by inhibiting theNEK7/NLRP3 signaling pathway.	Zhou, X et al. (67)
Synoviolin knockout mice	GSDMD	Synoviolin protected against periodontitis by regulating GSDMD	Pang, Y et al. (68)

### 4.4 TLR9

Periodontitis is a common chronic disease, and its progression may be regulated by interactions between host immunity and periodontal pathogens (151, 152). TLRs are type I transmembrane glycoproteins that belong to the pattern recognition receptor (PRR) class, which recognizes highly conserved structures on the surface of a large number of microorganisms and activates intrinsic immune cells through intercellular signaling pathways, thereby triggering an acquired immune response (153, 154).In recent years, many studies have revealed the ability of TLRs to recognize periodontal pathogens and modulate host innate immune responses to periodontal bacteria, including plasma membrane-associated TLR2, TLR4, and more recently the intrinsic intracellular sensor TLR9 (74, 76, 78, 155). *In vivo* evidence shows that TLR9-deficient (TLR9/) mice are resistant to periodontitis. This provides the first conceptual evidence for the involvement of nucleic acid sensors in periodontitis (101). In addition, TLR9 has been shown to modulate inflammation triggered by TLR2 and TLR4, suggesting possible crosstalk between these sensors during periodontal inflammation via downstream signaling pathways. Recent data suggest that TLR9 is one of the most up-regulated PRRs expressed in chronic periodontitis (156). DNA of the periodontal pathogen Porphyromonas gingivalis promotes its virulence in periodontitis by expressing inflammatory cytokines via the TLR9 signaling pathway (80, 156). TLR9 is present in gingival tissues, and nucleic acids significantly upregulate TLR9 gene expression in patients with periodontitis (76). TLRs signal through two pathways: a MyD88-dependent pathway and a MyD88-independent pathway.TLR9 recognizes viral nucleic acids through a MyD88-dependent pathway. The TLR9 signaling pathway activates the transcription factor NF-κB through inhibition of the nuclear factor-κB (i -κB) kinase (IKK) complex, leading to enhanced NF-κB signaling (157, 158). TLR9 is also present in macrophages and can sense bacterial DNA. in addition the distribution of TLR9 haplotypes and TLR9 (T1486C) genotypes may be associated with chronic periodontitis (75, 159). To model chronic periodontitis, TLR9 knockout mice or wild-type mice were exposed to Porphyromonas gingivalis. The data showed that bone loss was increased in wild-type mice compared with controls (not infected with Porphyromonas gingivalis), and no bone loss was detected in TLR9 knockout mice compared with controls, suggesting that alveolar bone loss in patients with periodontitis may be regulated by TLR9 (74). TLR9 plays a key role in periodontitis, and aberrant expression of TLR9 can be observed in patients with periodontitis, thus TLR9 has the potential to serve as a diagnostic or prognostic biomarker for periodontitis. Understanding the mechanisms by which TLR9 promotes periodontal inflammation could provide important insights into how to control aberrant periodontal inflammation as well as identify therapeutic targets and disease biomarkers that are critical for local and systemic outcomes (**Table 3**).

This work demonstrates the dynamic evolutionary process and structural relationship between the fields related to periodontitis and aging by knowledge mapping and data visualization, and preliminarily analyzes the research frontiers in this field. This study will provide more theoretical basis for researchers in this field. In conclusion, we should place greater emphasis on research regarding the association between periodontal disease and aging. Additionally, it is important to strengthen communication and collaboration among research institutions to facilitate the development of this field.

## Data availability statement

The raw data supporting the conclusions of this article will be made available by the authors, without undue reservation.

## Author contributions

XL: Conceptualization, Data curation, Formal analysis,Investigation, Methodology, Software, Visualization, Writing –original draft, Writing – review & editing. HL: Funding acquisition, Supervision, Writing – review & editing.

## Supplementary material

Supplementary Table 1 Search strategy.

Supplementary Table 2 National communications scale.

Supplementary Table 3 Institutional issuance scale.

Supplementary Table 4 Scale of Journal Publications.

Supplementary Figure 1 Double stacked diagram of journals.

Supplementary Table 5 Table of Journal Commonly Cited.

Supplementary Table 6 Table of authors’ publications and top 10 co-cited authors.

## References

[1] Ebersole JL Kirakodu SS Neumann E Orraca L Gonzalez Martinez J Gonzalez OA. Oral microbiome and gingival tissue apoptosis and autophagy transcriptomics. Front Immunol. (2020) 11:585414. doi: 10.3389/fimmu.2020.585414 33193408 PMC7604357

[2] Eke PI Borgnakke WS Genco RJ. Recent epidemiologic trends in periodontitis in the USA. Periodontol 2000. (2020) 82:257–67. doi: 10.1111/prd.12323 31850640

[3] Chen M Cai W Zhao S Shi L Chen Y Li X. Oxidative stress-related biomarkers in saliva and gingival crevicular fluid associated with chronic periodontitis: A systematic review and meta-analysis. J Clin Periodontol. (2019) 46:608–22. doi: 10.1111/jcpe.13112 30989678

[4] Parameter on chronic periodontitis with advanced loss of periodontal support. J Periodontol. (2000) 71 Suppl 5S:856–8. doi: 10.1902/jop.2000.71.5-S.856 29537504

[5] Valenti C Pagano S Bozza S Ciurnella E Lomurno G Capobianco B. Use of the er : YAG laser in conservative dentistry: evaluation of the microbial population in carious lesions. Materials (Basel). (2021) 14. doi: 10.3390/ma14092387 PMC8124663 34064339

[6] Ortensi L Sigari G La Rosa GRM Ferri A Grande F Pedull E. Digital planning of composite customized veneers using Digital Smile Design: Evaluation of its accuracy and manufacturing. Clin Exp Dent Res. (2022) 8:537–43. doi: 10.1002/cre2.570 PMC9033542 35362247

[7] Fulop T Larbi A Pawelec G Khalil A Cohen AA Hirokawa K. Immunology of aging: the birth of inflammaging. Clin Rev Allergy Immunol. (2023) 64:109–22. doi: 10.1007/s12016-021-08899-6 PMC8449217 34536213

[8] Di Micco R Krizhanovsky V Baker D d’Adda di Fagagna F. Cellular senescence in ageing: from mechanisms to therapeutic opportunities. Nat Rev Mol Cell Biol. (2021) 22:75–95. doi: 10.1038/s41580-020-00314-w 33328614 PMC8344376

[9] Ebersole JL Graves CL Gonzalez OA Dawson D 3rd Morford LA Huja PE. Aging, inflammation, immunity and periodontal disease. Periodontol 2000. (2016) 72:54–75. doi: 10.1111/prd.12135 27501491

[10] Eke PI Dye BA Wei L Thornton-Evans GO Genco RJ. Prevalence of periodontitis in adults in the United States: 2009 and 2010. J Dent Res. (2012) 91:914–20. doi: 10.1177/0022034512457373 22935673

[11] Bertl K Tangl S Rybaczek T Berger B Traindl-Prohazka M Schuller-Götzburg P. Prevalence and severity of periodontal disease in a historical Austrian population. J Periodontal Res. (2020) 55:931–45. doi: 10.1111/jre.12785 PMC7689777 32658361

[12] Mack F Mojon P Budtz-Jørgensen E Kocher T Splieth C Schwahn C. Caries and periodontal disease of the elderly in Pomerania, Germany: results of the Study of Health in Pomerania. Gerodontology. (2004) 21:27–36. doi: 10.1046/j.1741-2358.2003.00001.x 15074537

[13] Baima G Romandini M Citterio F Romano F Aimetti M. Periodontitis and accelerated biological aging: A geroscience approach. J Dent Res. (2022) 101:125–32. doi: 10.1177/00220345211037977 34609209

[14] Albuquerque-Souza E Crump KE Rattanaprukskul K Li Y Shelling B Xia-Juan X. TLR9 mediates periodontal aging by fostering senescence and inflammaging. J Dent Res. (2022) 101:1628–36. doi: 10.1177/00220345221110108 PMC9703528 35918888

[15] LL.ez-Ot0. C Blasco MA Partridge L Serrano M Kroemer G. Hallmarks of aging: An expanding universe. Cell. (2023) 186:243–78. doi: 10.1016/j.cell.2022.11.001 36599349

[16] Demontiero O Vidal C Duque G. Aging and bone loss: new insights for the clinician. Ther Adv Musculoskelet Dis. (2012) 4:61–76. doi: 10.1177/1759720x11430858 22870496 PMC3383520

[17] Franceschi C Campisi J. Chronic inflammation (inflammaging) and its potential contribution to age-associated diseases. J Gerontol A Biol Sci Med Sci. (2014) 69 Suppl 1:S4–9. doi: 10.1093/gerona/glu057 24833586

[18] Huttner EA MaChado DC de Oliveira RB Antunes AG Hebling E. Effects of human aging on periodontal tissues. Spec Care Dentist. (2009) 29:149–55. doi: 10.1111/j.1754-4505.2009.00082.x 19573041

[19] Hajishengallis G. Too old to fight? Aging and its toll on innate immunity. Mol Oral Microbiol. (2010) 25:25–37. doi: 10.1111/j.2041-1014.2009.00562.x 20305805 PMC2839454

[20] Boutin S Hagenfeld D Zimmermann H El Sayed N Höpker T Greiser HK. Clustering of subgingival microbiota reveals microbial disease ecotypes associated with clinical stages of periodontitis in a cross-sectional study. Front Microbiol. (2017) 8:340. doi: 10.3389/fmicb.2017.00340 28298910 PMC5331054

[21] Van Dyke TE. Shifting the paradigm from inhibitors of inflammation to resolvers of inflammation in periodontitis. J Periodontol. (2020) 91 Suppl 1:S19–s25. doi: 10.1002/jper.20-0088 32441774 PMC8142079

[22] Franceschi C Bonafe M Valensin S Olivieri F De Luca M Ottaviani E. Inflamm-aging. An evolutionary perspective on immunosenescence. Ann N Y Acad Sci. (2000) 908:244–54. doi: 10.1111/j.1749-6632.2000.tb06651.x 10911963

[23] Thompson DF Walker CK. A descriptive and historical review of bibliometrics with applications to medical sciences. Pharmacotherapy. (2015) 35:551–9. doi: 10.1002/phar.1586 25940769

[24] Akmal M Hasnain N Rehan A Iqbal U Hashmi S Fatima K. Glioblastome multiforme: A bibliometric analysis. World Neurosurg. (2020) 136:270–82. doi: 10.1016/j.wneu.2020.01.027 31953095

[25] Ma C Su H Li H. Global research trends on prostate diseases and erectile dysfunction: A bibliometric and visualized study. Front Oncol. (2020) 10:627891. doi: 10.3389/fonc.2020.627891 33643922 PMC7908828

[26] Wang S Zhou H Zheng L Zhu W Zhu L Feng D. Global trends in research of macrophages associated with acute lung injury over past 10 years: A bibliometric analysis. Front Immunol. (2021) 12:669539. doi: 10.3389/fimmu.2021.669539 34093568 PMC8173163

[27] Liu T Chen D Tang S Zou Z Yang F Zhang Y. P53 alleviates the progression of periodontitis by reducing M1-type macrophage differentiation. Inflammation. (2024). doi: 10.1007/s10753-024-01968-w PMC11343802 38319542

[28] Chen MH Wang YH Sun BJ Yu LM Chen QQ Han XX. HIF-1, activator DMOG inhibits alveolar bone resorption in murine periodontitis by regulating macrophage polarization. Int Immunopharmacol. (2021) 99:107901. doi: 10.1016/j.intimp.2021.107901 34273637

[29] Han Y Huang Y Gao P Yang Q Jia L Zheng Y. Leptin aggravates periodontitis by promoting M1 polarization via NLRP3. J Dent Res. (2022) 101:675–85. doi: 10.1177/00220345211059418 35050801

[30] Yan X Yuan Z Bian Y Jin L Mao Z Lei J. Uncoupling protein-2 regulates M1 macrophage infiltration of gingiva with periodontitis. Cent Eur J Immunol. (2020) 45:9–21. doi: 10.5114/ceji.2020.94664 32425675 PMC7226558

[31] Hou L Ye Y Gou H Tang H Zhou Y Xu X. A20 inhibits periodontal bone resorption and NLRP3-mediated M1 macrophage polarization. Exp Cell Res. (2022) 418:113264. doi: 10.1016/j.yexcr.2022.113264 35714941

[32] Wang Y Zhang X Wang J Zhang Y Ye Q Wang Y. Inflammatory Periodontal Ligament Stem Cells Drive M1 Macrophage Polarization via Exosomal miR-143-3p-Mediated Regulation of PI3K/AKT/NF-ed Signaling. Stem Cells. (2023) 41:184–99. doi: 10.1093/stmcls/sxac087 36520505

[33] Cui Y Hong S Xia Y Li X He X Hu X. Melatonin engineering M2 macrophage-derived exosomes mediate endoplasmic reticulum stress and immune reprogramming for periodontitis therapy. Adv Sci (Weinh). (2023) 10:e2302029. doi: 10.1002/advs.202302029 37452425 PMC10520618

[34] Nakao Y Fukuda T Zhang Q Sanui T Shinjo T Kou X. Exosomes from TNF-omes,2/a human gingiva-derived MSCs enhance M2 macrophage polarization and inhibit periodontal bone loss. Acta Biomater. (2021) 122:306–24. doi: 10.1016/j.actbio.2020.12.046 PMC7897289 33359765

[35] He XT Li X Zhang M Tian BM Sun LJ Bi CS. Role of molybdenum in material immunomodulation and periodontal wound healing: Targeting immunometabolism and mitochondrial function for macrophage modulation. Biomaterials. (2022) 283:121439. doi: 10.1016/j.biomaterials.2022.121439 35247634

[36] Liu J Wang H Zhang L Li X Ding X Ding G. Periodontal ligament stem cells promote polarization of M2 macrophages. J Leukoc Biol. (2022) 111:1185–97. doi: 10.1002/jlb.1ma1220-853rr 34982483

[37] Chen X Wan Z Yang L Song S Fu Z Tang K. Exosomes derived from reparative M2-like macrophages prevent bone loss in murine periodontitis models via IL-10 mRNA. J Nanobiotechnol. (2022) 20:110. doi: 10.1186/s12951-022-01314-y PMC8898524 35248085

[38] Liao Y Yan Q Cheng T Yao H Zhao Y Fu D. Sulforaphene Inhibits Periodontitis through Regulating Macrophage Polarization via Upregulating Dendritic Cell Immunoreceptor. J Agric Food Chem. (2023) 71:15538–52. doi: 10.1021/acs.jafc.3c02619 37823224

[39] Huang H Pan W Wang Y Kim HS Shao D Huang B. Nanoparticulate cell-free DNA scavenger for treating inflammatory bone loss in periodontitis. Nat Commun. (2022) 13:5925. doi: 10.1038/s41467-022-33492-6 36207325 PMC9546917

[40] Yang L Tao W Xie C Chen Q Zhao Y Zhang L. Interleukin-37 ameliorates periodontitis development by inhibiting NLRP3 inflammasome activation and modulating M1/M2 macrophage polarization. J Periodontal Res. (2024) 59:128–39. doi: 10.1111/jre.13196 37947055

[41] Guo X Huang Z Ge Q Yang L Liang D Huang Y. Glipizide alleviates periodontitis pathogenicity via inhibition of angiogenesis, osteoclastogenesis and M1/M2 macrophage ratio in periodontal tissue. Inflammation. (2023) 46:1917–31. doi: 10.1007/s10753-023-01850-1 37289398

[42] Wang Y Li C Wan Y Qi M Chen Q Sun Y. Quercetin-Loaded Ceria Nanocomposite Potentiate Dual-Directional Immunoregulation via Macrophage Polarization against Periodontal Inflammation. Small. (2021) 17:e2101505. doi: 10.1002/smll.202101505 34499411

[43] Deng J Golub LM Lee HM Bhatt HD Johnson F Xu TM. A novel modified-curcumin 2.24 resolves inflammation by promoting M2 macrophage polarization. Sci Rep. (2023) 13:15513. doi: 10.1038/s41598-023-42848-x 37726411 PMC10509274

[44] Yuan G Fu C Yang ST Yuh DY Hajishengallis G Yang S. RGS12 drives macrophage activation and osteoclastogenesis in periodontitis. J Dent Res. (2022) 101:448–57. doi: 10.1177/00220345211045303 PMC8935576 34796776

[45] Liu L Guo S Shi W Liu Q Huo F Wu Y. Bone marrow mesenchymal stem cell-derived small extracellular vesicles promote periodontal regeneration. Tissue Eng Part A. (2021) 27:962–76. doi: 10.1089/ten.TEA.2020.0141 32962564

[46] Li X Zhou R Han Y Zeng J Shi L Mao Y. Silibinin attenuates experimental periodontitis by downregulation of inflammation and oxidative stress. Oxid Med Cell Longev. (2023) 2023:5617800. doi: 10.1155/2023/5617800 36846719 PMC9946757

[47] Xiong Y Zhao B Zhang W Jia L Zhang Y Xu X. Curcumin promotes osteogenic differentiation of periodontal ligament stem cells through the PI3K/AKT/Nrf2 signaling pathway. Iran J Basic Med Sci. (2020) 23:954–60. doi: 10.22038/ijbms.2020.44070.10351 PMC7395181 32774819

[48] Bhattarai G Poudel SB Kook SH Lee JC. Resveratrol prevents alveolar bone loss in an experimental rat model of periodontitis. Acta Biomater. (2016) 29:398–408. doi: 10.1016/j.actbio.2015.10.031 26497626

[49] Jia L Xiong Y Zhang W Ma X Xu X. Metformin promotes osteogenic differentiation and protects against oxidative stress-induced damage in periodontal ligament stem cells via activation of the Akt/Nrf2 signaling pathway. Exp Cell Res. (2020) 386:111717. doi: 10.1016/j.yexcr.2019.111717 31715142

[50] Wei Y Fu J Wu W Ma P Ren L Yi Z. Quercetin prevents oxidative stress-induced injury of periodontal ligament cells and alveolar bone loss in periodontitis. Drug Des Devel Ther. (2021) 15:3509–22. doi: 10.2147/dddt.S315249 PMC8366957 34408403

[51] Xin L Zhou F Zhang C Zhong W Xu S Jing X. Four-Octyl itaconate ameliorates periodontal destruction via Nrf2-dependent antioxidant system. Int J Oral Sci. (2022) 14:27. doi: 10.1038/s41368-022-00177-1 35637195 PMC9151820

[52] Liu X Hou Y Yang M Xin X Deng Y Fu R. N-acetyl-l-cysteine-derived carbonized polymer dots with ROS scavenging via keap1-nrf2 pathway regulate alveolar bone homeostasis in periodontitis. Adv Healthc Mater. (2023) 12:e2300890. doi: 10.1002/adhm.202300890 37279380

[53] Li J Li Y Pan S Zhang L He L Niu Y. Paeonol attenuates ligation-induced periodontitis in rats by inhibiting osteoclastogenesis via regulating Nrf2/NF-ngogenesi signaling pathway. Biochimie. (2019) 156:129–37. doi: 10.1016/j.biochi.2018.09.004 30213522

[54] Zhu C Zhao Y Wu X Qiang C Liu J Shi J. The therapeutic role of baicalein in combating experimental periodontitis with diabetes via Nrf2 antioxidant signaling pathway. J Periodontal Res. (2020) 55:381–91. doi: 10.1111/jre.12722 31854466

[55] Huang X Liu Y Shen H Fu T Guo Y Qiu S. Chlorogenic acid attenuates inflammation in LPS-induced Human gingival fibroblasts via CysLT1R/Nrf2/NLRP3 signaling. Int Immunopharmacol. (2022) 107:108706. doi: 10.1016/j.intimp.2022.108706 35313270

[56] Zhou J Shi P Ma R Xie X Zhao L Wang J. Notopterol inhibits the NF-ib pathway and activates the PI3K/akt/nrf2 pathway in periodontal tissue. J Immunol. (2023) 211:1516–25. doi: 10.4049/jimmunol.2200727 37819772

[57] Fan Q Zhou XH Wang TF Zeng FJ Liu X Gu Y. Effects of epigallocatechin-3-gallate on oxidative stress, inflammation, and bone loss in a rat periodontitis model. J Dent Sci. (2023) 18:1567–75. doi: 10.1016/j.jds.2023.02.019 PMC10548010 37799898

[58] Wang L Pu W Wang C Lei L Li H. Microtubule affinity regulating kinase 4 promoted activation of the NLRP3 inflammasome-mediated pyroptosis in periodontitis. J Oral Microbiol. (2022) 14:2015130. doi: 10.1080/20002297.2021.2015130 34992737 PMC8725745

[59] Yang S Yin Y Sun Y Ai D Xia X Xu X. AZGP1 aggravates macrophage M1 polarization and pyroptosis in periodontitis. J Dent Res. (2024), 220345241235616. doi: 10.1177/00220345241235616 38491721

[60] Huang C Zhang C Yang P Chao R Yue Z Li C. Eldecalcitol inhibits LPS-induced NLRP3 inflammasome-dependent pyroptosis in human gingival fibroblasts by activating the nrf2/HO-1 signaling pathway. Drug Des Devel Ther. (2020) 14:4901–13. doi: 10.2147/dddt.S269223 PMC7671541 33223823

[61] Zhao D Wu Y Zhuang J Xu C Zhang F. Activation of NLRP1 and NLRP3 inflammasomes contributed to cyclic stretch-induced pyroptosis and release of IL-1a in human periodontal ligament cells. Oncotarget. (2016) 7:68292–302. doi: 10.18632/oncotarget.11944 PMC5356555 27626170

[62] He D Li X Zhang F Wang C Liu Y Bhawal UK. Dec2 inhibits macrophage pyroptosis to promote periodontal homeostasis. J Periodontal Implant Sci. (2022) 52:28–38. doi: 10.5051/jpis.2101380069 35187871 PMC8860764

[63] Gao Y Guo X Zhou Y Du J Lu C Zhang L. Kynurenic acid inhibits macrophage pyroptosis by suppressing ROS production via activation of the NRF2 pathway. Mol Med Rep. (2023) 28. doi: 10.3892/mmr.2023.13098 PMC10552067 37772394

[64] Peng Y Wang H Huang X Liu H Xiao J Wang C. Tet methylcytosine dioxygenase 1 modulates Porphyromonas gingivalis-triggered pyroptosis by regulating glycolysis in cementoblasts. Ann N Y Acad Sci. (2023) 1523:119–34. doi: 10.1111/nyas.14979 36934292

[65] Lv X Fan C Jiang Z Wang W Qiu X Ji Q. Isoliquiritigenin alleviates P. gingivalis-LPS/ATP-induced pyroptosis by inhibiting NF-ibi NLRP3/GSDMD signals in human gingival fibroblasts. Int Immunopharmacol. (2021) 101:108338. doi: 10.1016/j.intimp.2021.108338 34794890

[66] Chu Y Xu Y Yang W Chu K Li S Guo L. N-acetylcysteine protects human periodontal ligament fibroblasts from pyroptosis and osteogenic differentiation dysfunction through the SIRT1/NF-ontionetimp. signaling pathway. Arch Oral Biol. (2023) 148:105642. doi: 10.1016/j.archoralbio.2023.105642 36773561

[67] Zhou X Wang Q Nie L Zhang P Zhao P Yuan Q. Metformin ameliorates the NLPP3 inflammasome mediated pyroptosis by inhibiting the expression of NEK7 in diabetic periodontitis. Arch Oral Biol. (2020) 116:104763. doi: 10.1016/j.archoralbio.2020.104763 32480011

[68] Pang Y Liu L Wu S Wang J Liu L. Synoviolin alleviates GSDMD-mediated periodontitis by suppressing its stability. Immun Inflammation Dis. (2023) 11:e880. doi: 10.1002/iid3.880 PMC10336677 37506160

[69] Yumoto H Chou HH Takahashi Y Davey M Gibson FC 3rd Genco CA. Sensitization of human aortic endothelial cells to lipopolysaccharide via regulation of Toll-like receptor 4 by bacterial fimbria-dependent invasion. Infect Immun. (2005) 73:8050–9. doi: 10.1128/iai.73.12.8050-8059.2005 PMC1307031 16299299

[70] Sun Y Shu R Li CL Zhang MZ. Gram-negative periodontal bacteria induce the activation of Toll-like receptors 2 and 4, and cytokine production in human periodontal ligament cells. J Periodontol. (2010) 81:1488–96. doi: 10.1902/jop.2010.100004 20528699

[71] Wara-aswapati N Chayasadom A Surarit R Pitiphat W Boch JA Nagasawa T. Induction of toll-like receptor expression by Porphyromonas gingivalis. J Periodontol. (2013) 84:1010–8. doi: 10.1902/jop.2012.120362 23003918

[72] Zhang Y Li X. Lipopolysaccharide-regulated production of bone sialoprotein and interleukin-8 in human periodontal ligament fibroblasts: the role of toll-like receptors 2 and 4 and the MAPK pathway. J Periodontal Res. (2015) 50:141–51. doi: 10.1111/jre.12193 24854880

[73] Kajita K Honda T Amanuma R Domon H Okui T Ito H. Quantitative messenger RNA expression of Toll-like receptors and interferon-alpha1 in gingivitis and periodontitis. Oral Microbiol Immunol. (2007) 22:398–402. doi: 10.1111/j.1399-302X.2007.00377.x 17949343

[74] Kim PD Xia-Juan X Crump KE Abe T Hajishengallis G Sahingur SE. Toll-like receptor 9-mediated inflammation triggers alveolar bone loss in experimental murine periodontitis. Infect Immun. (2015) 83:2992–3002. doi: 10.1128/iai.00424-15 25964477 PMC4468549

[75] Holla LI Vokurka J Hrdlickova B Augustin P Fassmann A. Association of Toll-like receptor 9 haplotypes with chronic periodontitis in Czech population. J Clin Periodontol. (2010) 37:152–9. doi: 10.1111/j.1600-051X.2009.01523.x 20041977

[76] Sahingur SE Xia XJ Voth SC Yeudall WA Gunsolley JC. Increased nucleic Acid receptor expression in chronic periodontitis. J Periodontol. (2013) 84:e48–57. doi: 10.1902/jop.2013.120739 23646855

[77] Chen YC Liu CM Jeng JH Ku CC. Association of pocket epithelial cell proliferation in periodontitis with TLR9 expression and inflammatory response. J Formos Med Assoc. (2014) 113:549–56. doi: 10.1016/j.jfma.2012.07.043 25037760

[78] Marchesan J Jiao Y Schaff RA Hao J Morelli T Kinney JS. TLR4, NOD1 and NOD2 mediate immune recognition of putative newly identified periodontal pathogens. Mol Oral Microbiol. (2016) 31:243–58. doi: 10.1111/omi.12116 PMC4713362 26177212

[79] Cecil JD O’Brien-Simpson NM Lenzo JC Holden JA Chen YY Singleton W. Differential responses of pattern recognition receptors to outer membrane vesicles of three periodontal pathogens. PloS One. (2016) 11:e0151967. doi: 10.1371/journal.pone.0151967 27035339 PMC4818014

[80] Edfeldt K Swedenborg J Hansson GK Yan ZQ. Expression of toll-like receptors in human atherosclerotic lesions: a possible pathway for plaque activation. Circulation. (2002) 105:1158–61. 11889007

[81] Latz E Schoenemeyer A Visintin A Fitzgerald KA Monks BG Knetter CF. TLR9 signals after translocating from the ER to CpG DNA in the lysosome. Nat Immunol. (2004) 5:190–8. doi: 10.1038/ni1028 14716310

[82] González OA Li M Ebersole JL Huang CB. HIV-1 reactivation induced by the periodontal pathogens Fusobacterium nucleatum and Porphyromonas gingivalis involves Toll-like receptor 2 [corrected] and 9 activation in monocytes/macrophages. Clin Vaccine Immunol. (2010) 17:1417–27. doi: 10.1128/cvi.00009-10 PMC2944464 20610663

[83] Liu G Jiang R Jin Y. Sciatic nerve injury repair: a visualized analysis of research fronts and development trends. Neural Regener Res. (2014) 9:1716–22. doi: 10.4103/1673-5374.141810 PMC4211194 25374595

[84] Xiao F Li C Sun J Zhang L. Knowledge domain and emerging trends in organic photovoltaic technology: A scientometric review based on citeSpace analysis. Front Chem. (2017) 5:67. doi: 10.3389/fchem.2017.00067 28966923 PMC5605557

[85] Ma L Ma J Teng M Li Y. Visual analysis of colorectal cancer immunotherapy: A bibliometric analysis from 2012 to 2021. Front Immunol. (2022) 13:843106. doi: 10.3389/fimmu.2022.843106 35432385 PMC9009266

[86] Graves D. Cytokines that promote periodontal tissue destruction. J Periodontol. (2008) 79:1585–91. doi: 10.1902/jop.2008.080183 18673014

[87] Sczepanik FSC Grossi ML Casati M Goldberg M Glogauer M Fine N. Periodontitis is an inflammatory disease of oxidative stress: We should treat it that way. Periodontol 2000. (2020) 84:45–68. doi: 10.1111/prd.12342 32844417

[88] Clark D Kotronia E Ramsay SE. Frailty, aging, and periodontal disease: Basic biologic considerations. Periodontol 2000. (2021) 87:143–56. doi: 10.1111/prd.12380 PMC8771712 34463998

[89] Tan J Dai A Pan L Zhang L Wang Z Ke T. Inflamm-aging-related cytokines of IL-17 and IFN-7 accelerate osteoclastogenesis and periodontal destruction. J Immunol Res. (2021) 2021:9919024. doi: 10.1155/2021/9919024 34395635 PMC8357511

[90] Scannapieco FA Cantos A. Oral inflammation and infection, and chronic medical diseases: implications for the elderly. Periodontol 2000. (2016) 72:153–75. doi: 10.1111/prd.12129 27501498

[91] Hajishengallis G. Aging and its impact on innate immunity and inflammation: implications for periodontitis. J Oral Biosci. (2014) 56:30–7. doi: 10.1016/j.job.2013.09.001 PMC3974203 24707191

[92] Krabbe KS Pedersen M Bruunsgaard H. Inflammatory mediators in the elderly. Exp Gerontol. (2004) 39:687–99. doi: 10.1016/j.exger.2004.01.009 15130663

[93] LeMaoult J Szabo P Weksler ME. Effect of age on humoral immunity, selection of the B-cell repertoire and B-cell development. Immunol Rev. (1997) 160:115–26. doi: 10.1111/j.1600-065x.1997.tb01032.x 9476670

[94] Schou S Holmstrup P Kornman KS. Non-human primates used in studies of periodontal disease pathogenesis: a review of the literature. J Periodontol. (1993) 64:497–508. doi: 10.1902/jop.1993.64.6.497 8336250

[95] Feng X McDonald JM. Disorders of bone remodeling. Annu Rev Pathol. (2011) 6:121–45. doi: 10.1146/annurev-pathol-011110-130203 PMC3571087 20936937

[96] Ogura N Matsuda U Tanaka F Shibata Y Takiguchi H Abiko Y. *In vitro* senescence enhances IL-6 production in human gingival fibroblasts induced by lipopolysaccharide from Campylobacter rectus. Mech Ageing Dev. (1996) 87:47–59. doi: 10.1016/0047-6374(96)01701-0 8735906

[97] Chen LP Chiang CK Chan CP Hung KY Huang CS. Does periodontitis reflect inflammation and malnutrition status in hemodialysis patients? Am J Kidney Dis. (2006) 47:815–22. doi: 10.1053/j.ajkd.2006.01.018 16632020

[98] Sanz M Del Castillo AM Jepsen S Gonzalez-Juanatey JR D’Aiuto F Bouchard P. Periodontitis and cardiovascular diseases. Consensus report. Glob Heart. (2020) 15:1. doi: 10.5334/gh.400 32489774 PMC7218770

[99] Kinane DF Stathopoulou PG Papapanou PN. Periodontal diseases. Nat Rev Dis Primers. (2017) 3:17038. doi: 10.1038/nrdp.2017.38 28805207

[100] D’Aiuto F Parkar M Andreou G Suvan J Brett PM Ready D. Periodontitis and systemic inflammation: control of the local infection is associated with a reduction in serum inflammatory markers. J Dent Res. (2004) 83:156–60. doi: 10.1177/154405910408300214 14742655

[101] Hajishengallis G. Interconnection of periodontal disease and comorbidities: Evidence, mechanisms, and implications. Periodontol 2000. (2022) 89:9–18. doi: 10.1111/prd.12430 35244969 PMC9018559

[102] Pink C Kocher T Meisel P Dörr M Markus MR Jablonowski L. Longitudinal effects of systemic inflammation markers on periodontitis. J Clin Periodontol. (2015) 42:988–97. doi: 10.1111/jcpe.12473 26472626

[103] Sharma P Fenton A Dias IHK Heaton B Brown CLR Sidhu A. Oxidative stress links periodontal inflammation and renal function. J Clin Periodontol. (2021) 48:357–67. doi: 10.1111/jcpe.13414 PMC7986430 33368493

[104] Sari A Davutoglu V Bozkurt E Taner IL Erciyas K. Effect of periodontal disease on oxidative stress markers in patients with atherosclerosis. Clin Oral Investig. (2022) 26:1713–24. doi: 10.1007/s00784-021-04144-8 34415433

[105] Rodrigues LP Teixeira VR Alencar-Silva T Simonassi-Paiva B Pereira RW Pogue R. Hallmarks of aging and immunosenescence: Connecting the dots. Cytokine Growth Factor Rev. (2021) 59:9–21. doi: 10.1016/j.cytogfr.2021.01.006 33551332

[106] Martinez FO Gordon S. The M1 and M2 paradigm of macrophage activation: time for reassessment. F1000Prime Rep. (2014) 6:13. doi: 10.12703/p6-13 24669294 PMC3944738

[107] Gordon S. Alternative activation of macrophages. Nat Rev Immunol. (2003) 3:23–35. doi: 10.1038/nri978 12511873

[108] Zhuang Z Yoshizawa-Smith S Glowacki A Maltos K Pacheco C Shehabeldin M. Induction of M2 macrophages prevents bone loss in murine periodontitis models. J Dent Res. (2019) 98:200–8. doi: 10.1177/0022034518805984 PMC6761736 30392438

[109] Almubarak A Tanagala KKK Papapanou PN Lalla E Momen-Heravi F. Disruption of monocyte and macrophage homeostasis in periodontitis. Front Immunol. (2020) 11:330. doi: 10.3389/fimmu.2020.00330 32210958 PMC7067288

[110] Sloniak MC Lepique AP Nakao LYS Villar CC. Alterations in macrophage polarization play a key role in control and development of periodontal diseases. J Indian Soc Periodontol. (2023) 27:578–82. doi: 10.4103/jisp.jisp_75_23 PMC10906788 38434507

[111] Yamamoto M Fujihashi K Hiroi T McGhee JR Van Dyke TE Kiyono H. Molecular and cellular mechanisms for periodontal diseases: role of Th1 and Th2 type cytokines in induction of mucosal inflammation. J Periodontal Res. (1997) 32:115–9. doi: 10.1111/j.1600-0765.1997.tb01391.x 9085220

[112] Yang J Zhu Y Duan D Wang P Xin Y Bai L. Enhanced activity of macrophage M1/M2 phenotypes in periodontitis. Arch Oral Biol. (2018) 96:234–42. doi: 10.1016/j.archoralbio.2017.03.006 28351517

[113] Wang W Zheng C Yang J Li B. Intersection between macrophages and periodontal pathogens in periodontitis. J Leukoc Biol. (2021) 110:577–83. doi: 10.1002/jlb.4mr0421-756r 34028883

[114] Mosser DM Edwards JP. Exploring the full spectrum of macrophage activation. Nat Rev Immunol. (2008) 8:958–69. doi: 10.1038/nri2448 PMC2724991 19029990

[115] Li W Zhang Z Wang ZM. Differential immune cell infiltrations between healthy periodontal and chronic periodontitis tissues. BMC Oral Health. (2020) 20:293. doi: 10.1186/s12903-020-01287-0 33109155 PMC7590666

[116] Zhou LN Bi CS Gao LN An Y Chen F Chen FM. Macrophage polarization in human gingival tissue in response to periodontal disease. Oral Dis. (2019) 25:265–73. doi: 10.1111/odi.12983 30285304

[117] Shapouri-Moghaddam A Mohammadian S Vazini H Taghadosi M Esmaeili SA Mardani F. Macrophage plasticity, polarization, and function in health and disease. J Cell Physiol. (2018) 233:6425–40. doi: 10.1002/jcp.26429 PMC13482560 29319160

[118] Atri C Guerfali FZ Laouini D. Role of human macrophage polarization in inflammation during infectious diseases. Int J Mol Sci. (2018) 19. doi: 10.3390/ijms19061801 PMC6032107 29921749

[119] Locati M Curtale G Mantovani A. Diversity, mechanisms, and significance of macrophage plasticity. Annu Rev Pathol. (2020) 15:123–47. doi: 10.1146/annurev-pathmechdis-012418-012718 PMC7176483 31530089

[120] Chen B Li S Chang Y Zhang J Liu J Dong Y. Macrophages contribute to periodontal wound healing mainly in the tissue proliferation stage. J Periodontal Res. (2023) 58:122–30. doi: 10.1111/jre.13074 36398469

[121] Ortega-Gga-Gr A Perretti M Soehnlein O. Resolution of inflammation: an integrated view. EMBO Mol Med. (2013) 5:661–74. doi: 10.1002/emmm.201202382 PMC3662311 23592557

[122] Sun X Gao J Meng X Lu X Zhang L Chen R. Polarized macrophages in periodontitis: characteristics, function, and molecular signaling. Front Immunol. (2021) 12:763334. doi: 10.3389/fimmu.2021.763334 34950140 PMC8688840

[123] Gibon E Loi F Córdova LA Pajarinen J Lin T Lu L. Aging affects bone marrow macrophage polarization: relevance to bone healing. Regener Eng Transl Med. (2016) 2:98–104. doi: 10.1007/s40883-016-0016-5 PMC5270653 28138512

[124] Zhou F Wang Z Zhang G Wu Y Xiong Y. Immunosenescence and inflammaging: Conspiracies against alveolar bone turnover. Oral Dis. (2023). doi: 10.1111/odi.14642 37288702

[125] Pihlstrom BL Michalowicz BS Johnson NW. Periodontal diseases. Lancet. (2005) 366:1809–20. doi: 10.1016/s0140-6736(05)67728-8 16298220

[126] Novakovii N Cakik S Todorovii T Raicevii BA Dozi; I Petrovii V. Antioxidative status of saliva before and after non-surgical periodontal treatment. Srp Arh Celok Lek. (2013) 141:163–8. doi: 10.2298/sarh1304163n 23745337

[127] Ling MR Chapple IL Matthews JB. Neutrophil superoxide release and plasma C-reactive protein levels pre- and post-periodontal therapy. J Clin Periodontol. (2016) 43:652–8. doi: 10.1111/jcpe.12575 27168055

[128] Shin MS Shin HS Ahn YB Kim HD. Association between periodontitis and salivary 8-hydroxydeoxyguanosine among Korean rural adults. Community Dent Oral Epidemiol. (2016) 44:381–9. doi: 10.1111/cdoe.12225 26919660

[129] Ohnishi T Bandow K Kakimoto K Machigashira M Matsuyama T Matsuguchi T. Oxidative stress causes alveolar bone loss in metabolic syndrome model mice with type 2 diabetes. J Periodontal Res. (2009) 44:43–51. doi: 10.1111/j.1600-0765.2007.01060.x 18973548

[130] Chapple IL Matthews JB. The role of reactive oxygen and antioxidant species in periodontal tissue destruction. Periodontol 2000. (2007) 43:160–232. doi: 10.1111/j.1600-0757.2006.00178.x 17214840

[131] Ramesh A Varghese SS Doraiswamy JN Malaiappan S. Herbs as an antioxidant arsenal for periodontal diseases. J Intercult Ethnopharmacol. (2016) 5:92–6. doi: 10.5455/jice.20160122065556 PMC4805154 27069730

[132] Baňasová L Kamodyov4 N Janodyov4 K T.;odyo Ľ Stanko P Turnk J. Salivary DNA and markers of oxidative stress in patients with chronic periodontitis. Clin Oral Investig. (2015) 19:201–7. doi: 10.1007/s00784-014-1236-z 24677171

[133] Su H Gornitsky M Velly AM Yu H Benarroch M Schipper HM. Salivary DNA, lipid, and protein oxidation in nonsmokers with periodontal disease. Free Radic Biol Med. (2009) 46:914–21. doi: 10.1016/j.freeradbiomed.2009.01.008 19280702

[134] Konopka T Kr;o K Kopep W Gerber H. Total antioxidant status and 8-hydroxy-2’-deoxyguanosine levels in gingival and peripheral blood of periodontitis patients. Arch Immunol Ther Exp (Warsz). (2007) 55:417–22. doi: 10.1007/s00005-007-0047-1 PMC2766448 18060366

[135] Yin KJ Huang JX Wang P Yang XK Tao SS Li HM. No genetic causal association between periodontitis and arthritis: A bidirectional two-sample mendelian randomization analysis. Front Immunol. (2022) 13:808832. doi: 10.3389/fimmu.2022.808832 35154127 PMC8825874

[136] Bartold PM Marshall RI Haynes DR. Periodontitis and rheumatoid arthritis: a review. J Periodontol. (2005) 76:2066–74. doi: 10.1902/jop.2005.76.11-S.2066 16277578

[137] Boyce BF Xing L. Biology of RANK, RANKL, and osteoprotegerin. Arthritis Res Ther. (2007) 9 Suppl 1:S1. doi: 10.1186/ar2165 17634140 PMC1924516

[138] Belibasakis GN Bostanci N. The RANKL-OPG system in clinical periodontology. J Clin Periodontol. (2012) 39:239–48. doi: 10.1111/j.1600-051X.2011.01810.x 22092994

[139] Baltaccltac E Yuva P Ayda,c G Alver A Kahraman C Karabulut E. Lipid peroxidation levels and total oxidant/antioxidant status in serum and saliva from patients with chronic and aggressive periodontitis. Oxidative stress index: a new biomarker for periodontal disease? J Periodontol. (2014) 85:1432–41. doi: 10.1902/jop.2014.130654 24635543

[140] Ikeda E Ikeda Y Wang Y Fine N Sheikh Z Viniegra A. Resveratrol derivative-rich melinjo seed extract induces healing in a murine model of established periodontitis. J Periodontol. (2018) 89:586–95. doi: 10.1002/jper.17-0352 29856488

[141] Chiu AV Saigh MA McCulloch CA Glogauer M. The role of nrF2 in the regulation of periodontal health and disease. J Dent Res. (2017) 96:975–83. doi: 10.1177/0022034517715007 28617616

[142] Vo TTT Chu PM Tuan VP Te JS Lee IT. The promising role of antioxidant phytochemicals in the prevention and treatment of periodontal disease via the inhibition of oxidative stress pathways: updated insights. Antioxid (Basel). (2020) 9. doi: 10.3390/antiox9121211 PMC7760335 33271934

[143] Kanzaki H Wada S Narimiya T Yamaguchi Y Katsumata Y Itohiya K. Pathways that regulate ROS scavenging enzymes, and their role in defense against tissue destruction in periodontitis. Front Physiol. (2017) 8:351. doi: 10.3389/fphys.2017.00351 28611683 PMC5447763

[144] Wei SJ Zhang Q Xiang YJ Peng LY Peng W Ren Q. Guizhi-Shaoyao-Zhimu decoction attenuates bone erosion in rats that have collagen-induced arthritis via modulating NF-ul signalling to suppress osteoclastogenesis. Pharm Biol. (2021) 59:262–74. doi: 10.1080/13880209.2021.1876100 PMC7906619 33626293

[145] Quirke AM Lugli EB Wegner N Hamilton BC Charles P Chowdhury M. Heightened immune response to autocitrullinated Porphyromonas gingivalis peptidylarginine deiminase: a potential mechanism for breaching immunologic tolerance in rheumatoid arthritis. Ann Rheum Dis. (2014) 73:263–9. doi: 10.1136/annrheumdis-2012-202726 PMC3888615 23463691

[146] Mei YM Li L Wang XQ Zhang M Zhu LF Fu YW. AGEs induces apoptosis and autophagy via reactive oxygen species in human periodontal ligament cells. J Cell Biochem. (2020) 121:3764–79. doi: 10.1002/jcb.29499 31680325

[147] Shi J Gao W Shao F. Pyroptosis: gasdermin-mediated programmed necrotic cell death. Trends Biochem Sci. (2017) 42:245–54. doi: 10.1016/j.tibs.2016.10.004 27932073

[148] Franco C Patricia HR Timo S Claudia B Marcela H. Matrix metalloproteinases as regulators of periodontal inflammation. Int J Mol Sci. (2017) 18. doi: 10.3390/ijms18020440 PMC5343974 28218665

[149] Bostanci N Emingil G Saygan B Turkoglu O Atilla G Curtis MA. Expression and regulation of the NALP3 inflammasome complex in periodontal diseases. Clin Exp Immunol. (2009) 157:415–22. doi: 10.1111/j.1365-2249.2009.03972.x PMC2745037 19664151

[150] Chen Q Liu X Wang D Zheng J Chen L Xie Q. Periodontal inflammation-triggered by periodontal ligament stem cell pyroptosis exacerbates periodontitis. Front Cell Dev Biol. (2021) 9:663037. doi: 10.3389/fcell.2021.663037 33869229 PMC8049442

[151] O’Brien-Simpson NM Pathirana RD Paolini RA Chen YY Veith PD Tam V. An immune response directed to proteinase and adhesin functional epitopes protects against Porphyromonas gingivalis-induced periodontal bone loss. J Immunol. (2005) 175:3980–9. doi: 10.4049/jimmunol.175.6.3980 16148146

[152] Sorsa T Tervahartiala T Leppilahti J Hernandez M Gamonal J Tuomainen AM. Collagenase-2 (MMP-8) as a point-of-care biomarker in periodontitis and cardiovascular diseases. Therapeutic response to non-antimicrobial properties of tetracyclines. Pharmacol Res. (2011) 63:108–13. doi: 10.1016/j.phrs.2010.10.005 20937384

[153] Flórez-Álvarez L Ruiz-Perez L Taborda N Hernandez JC. Toll-like receptors as a therapeutic target in cancer, infections and inflammatory diseases. Immunotherapy. (2020) 12:311–22. doi: 10.2217/imt-2019-0096 32237938

[154] Kumar V. Toll-like receptors in adaptive immunity. Handb Exp Pharmacol. (2022) 276:95–131. doi: 10.1007/164_2021_543 34510306

[155] Crump KE Sahingur SE. Microbial nucleic acid sensing in oral and systemic diseases. J Dent Res. (2016) 95:17–25. doi: 10.1177/0022034515609062 26438211 PMC4700663

[156] Crump KE Oakley JC Xia-Juan X Madu TC Devaki S Mooney EC. Interplay of toll-like receptor 9, myeloid cells, and deubiquitinase A20 in periodontal inflammation. Infect Immun. (2017) 85. doi: 10.1128/iai.00814-16 PMC5203663 27849177

[157] O’Neill LA. When signaling pathways collide: positive and negative regulation of toll-like receptor signal transduction. Immunity. (2008) 29:12–20. doi: 10.1016/j.immuni.2008.06.004 18631453

[158] Karapetyan L Luke JJ Davar D. Toll-like receptor 9 agonists in cancer. Onco Targets Ther. (2020) 13:10039–60. doi: 10.2147/ott.S247050 PMC7553670 33116588

[159] Sahingur SE Xia XJ Gunsolley J Schenkein HA Genco RJ De Nardin E. Single nucleotide polymorphisms of pattern recognition receptors and chronic periodontitis. J Periodontal Res. (2011) 46:184–92. doi: 10.1111/j.1600-0765.2010.01327.x 21118416

