# Supplementary material for: Global trends in research on aging associated with periodontitis from 2002 to 2023: a bibliometric analysis
Source: Front Endocrinol (Lausanne). 2024 May 10;15:1374027. doi: 10.3389/fendo.2024.1374027 (PMC11116588; doi:10.3389/fendo.2024.1374027)
Supplement: Supplementary Table 1 — Search strategy. [file Table_1.docx]

|  | Kewords | search formula |
| --- | --- | --- |
| Aging | Aging；Senescence；Biological Aging；Aging, Biological | (((TS=(aging)) OR TS=(Senescence)) OR TS=(Biological Aging)) OR TS=(Aging, Biological) |
| periodontitis | Periodontitis；Periodontitides；Pericementitis；Pericementitides | (((TS=(periodontitis)) OR TS=(Periodontitides)) OR TS=(Pericementitis)) OR TS=(Pericementitides) |

TableS1 search strategy
