# Supplementary material for: Global trends in research on aging associated with periodontitis from 2002 to 2023: a bibliometric analysis
Source: Front Endocrinol (Lausanne). 2024 May 10;15:1374027. doi: 10.3389/fendo.2024.1374027 (PMC11116588; doi:10.3389/fendo.2024.1374027)
Supplement: Supplementary Table 2 — National communications scale. [file Table_2.docx]

| Rank | Country/region | Article counts | Percentage (%) | Citation | Citation per publication |
| --- | --- | --- | --- | --- | --- |
| 1 | USA | 1024 | 23.02 | 41954 | 40.97 |
| 2 | BRAZIL | 403 | 9.06 | 9007 | 22.35 |
| 3 | CHINA | 372 | 8.36 | 6420 | 17.26 |
| 4 | JAPAN | 365 | 8.21 | 8148 | 22.32 |
| 5 | GERMANY | 349 | 7.85 | 9882 | 28.32 |
| 6 | SWEDEN | 293 | 6.59 | 9339 | 31.87 |
| 7 | ENGLAND | 255 | 5.73 | 14287 | 56.03 |
| 8 | SOUTH KOREA | 239 | 5.37 | 3742 | 15.66 |
| 9 | ITALY | 196 | 4.41 | 5245 | 26.76 |
| 10 | TURKEY | 159 | 3.57 | 2900 | 18.24 |

Table S2: National communications scale
