# Supplementary material for: Global trends in research on aging associated with periodontitis from 2002 to 2023: a bibliometric analysis
Source: Front Endocrinol (Lausanne). 2024 May 10;15:1374027. doi: 10.3389/fendo.2024.1374027 (PMC11116588; doi:10.3389/fendo.2024.1374027)
Supplement: Supplementary Table 3 — Institutional issuance scale. [file Table_3.docx]

| Rank | Institution | Country | Number of studies | Total citations | Average citation |
| --- | --- | --- | --- | --- | --- |
| 1 | Karolinska Inst | Sweden | 116 | 3351 | 21.86 |
| 2 | Univ Helsinki | Finland | 105 | 3315 | 57.56 |
| 3 | Univ Washington | USA | 86 | 8051 | 16.37 |
| 4 | Seoul Natl Univ | South Korea | 74 | 1226 | 34.60 |
| 5 | Columbia Univ | USA | 68 | 4174 | 27.13 |
| 6 | Univ Sao Paulo | Brazil | 68 | 1557 | 20.57 |
| 7 | Univ Bern | Sweden | 66 | 2691 | 11.23 |
| 8 | Univ N Carolina | USA | 56 | 3080 | 15.90 |
| 9 | Univ Michigan | USA | 54 | 3101 | 28.14 |
| 10 | Forsyth Inst | USA | 52 | 2144 | 30.00 |

Table S3. Institutional issuance scale
