# Supplementary material for: Global trends in research on aging associated with periodontitis from 2002 to 2023: a bibliometric analysis
Source: Front Endocrinol (Lausanne). 2024 May 10;15:1374027. doi: 10.3389/fendo.2024.1374027 (PMC11116588; doi:10.3389/fendo.2024.1374027)
Supplement: Supplementary Table 4 — Scale of Journal Publications. [file Table_4.docx]

| Rank | Journal | Article counts | Percentage（4448） | IF | Quartile in category |
| --- | --- | --- | --- | --- | --- |
| 1 | journal of periodontology | 522 | 11.74 | 4.3 | Q1 |
| 2 | journal of clinical periodontology | 461 | 10.36 | 6.7 | Q1 |
| 3 | journal of periodontal research | 166 | 3.73 | 3.5 | Q2 |
| 4 | clinical oral investigations | 138 | 3.10 | 3.4 | Q2 |
| 5 | bmc oral health | 118 | 2.65 | 2.9 | Q2 |
| 6 | journal of dental research | 109 | 2.45 | 7.6 | Q1 |
| 7 | journal of endodontics | 86 | 1.93 | 4.2 | Q1 |
| 8 | international endodontic journal | 75 | 1.69 | 5.0 | Q1 |
| 9 | plos one | 71 | 1.60 | 3.7 | Q2 |
| 10 | international journal of environmental research and public health | 66 | 1.48 | 4.6 | Q2 |

Table S4.Scale of Journal Publications
