# Supplementary figures and images for: Global trends in research on aging associated with periodontitis from 2002 to 2023: a bibliometric analysis
Source: Front Endocrinol (Lausanne). 2024 May 10;15:1374027. doi: 10.3389/fendo.2024.1374027 (PMC11116588; doi:10.3389/fendo.2024.1374027)

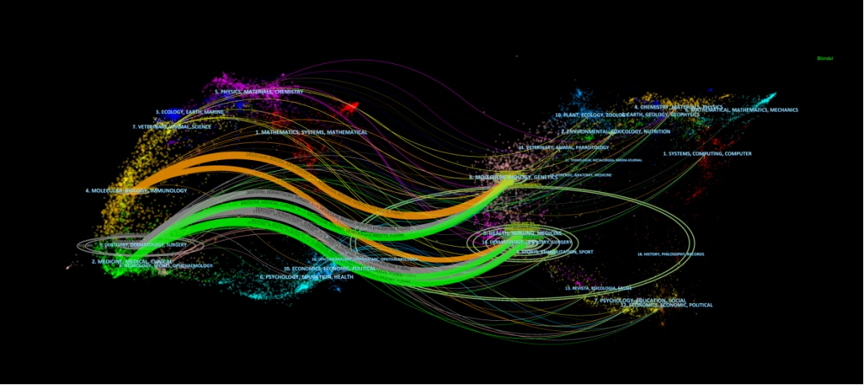

Supplement: Supplementary Figure 1 — Double stacked diagram of journals. [file Image_1.tif]
