# Supplementary material for: Global trends in research on aging associated with periodontitis from 2002 to 2023: a bibliometric analysis
Source: Front Endocrinol (Lausanne). 2024 May 10;15:1374027. doi: 10.3389/fendo.2024.1374027 (PMC11116588; doi:10.3389/fendo.2024.1374027)
Supplement: Supplementary Table 5 — Table of Journal Commonly Cited. [file Table_5.docx]

| Rank | Cited Journal | Citation | IF（2020） | Quartile in category |
| --- | --- | --- | --- | --- |
| 1 | J PERIODONTOL | 3601 | 4.3 | Q1 |
| 2 | J CLIN PERIODONTOL | 3466 | 6.7 | Q1 |
| 3 | J DENT RES | 2711 | 7.6 | Q1 |
| 4 | PERIODONTOL 2000 | 2252 | 18.6 | Q2 |
| 5 | J PERIODONTAL RES | 1922 | 3.5 | Q1 |
| 6 | J AM DENT ASSOC | 1096 | 3.9 | Q1 |
| 7 | LANCET | 1062 | 168.9 | Q1 |
| 8 | COMMUNITY DENT ORAL | 1050 | 2.3 | Q3 |
| 9 | PLOS ONE | 1018 | 3.7 | Q2 |
| 10 | ACTA ODONTOL SCAND | 989 | 2.0 | Q3 |

Table S5.Table of Journal Commonly Cited
