# Supplementary material for: Global trends in research on aging associated with periodontitis from 2002 to 2023: a bibliometric analysis
Source: Front Endocrinol (Lausanne). 2024 May 10;15:1374027. doi: 10.3389/fendo.2024.1374027 (PMC11116588; doi:10.3389/fendo.2024.1374027)
Supplement: Supplementary Table 6 — Table of authors’ publications and top 10 co-cited authors. [file Table_6.docx]

| Rank | Author | Count | Location | Rank | Co-cited author | Citation |
| --- | --- | --- | --- | --- | --- | --- |
| 1 | kocher, thomas | 50 | GERMANY | 1 | TONETTI MS | 698 |
| 2 | papapanou, panos n. | 31 | USA | 2 | EKE PI | 672 |
| 3 | holtfreter, birte | 28 | GERMANY | 3 | PAGE RC | 534 |
| 4 | sorsa, timo | 27 | FINLAND | 4 | ALBANDAR JM | 470 |
| 5 | kim, hyun-duck | 25 | KOREA | 5 | LOE H | 456 |
| 6 | morita, manabu | 25 | JAPAN | 6 | ARMITAGE G C | 440 |
| 7 | renvert, stefan | 24 | SWEDEN | 7 | PAPAPANOU PN | 432 |
| 8 | persson, g. rutger | 23 | USA | 8 | GENCO RJ | 422 |
| 9 | ekuni, daisuke | 22 | JAPAN | 9 | SOCRANSKY SS | 362 |
| 10 | botelho, joao | 21 | PORTUGAL | 10 | AINAMO J | 339 |

Table S6.Table of authors' publications and top 10 co-cited authors
